# Polyoxometalate-directed interfacial assembly of multifunctional mesoporous polydopamine nanomotors

**DOI:** 10.1093/nsr/nwag214

**Published:** 2026-04-07

**Authors:** Chunhong Chen, Lihua Wang, Yu Yang, Tengjin Wang, Ting Li, Hao Zhang, Ruizheng Liang, Lianhui Wang, Yong Wang

**Affiliations:** State Key Laboratory of Flexible Electronics (LoFE) & Institute of Advanced Materials (IAM), Nanjing University of Posts & Telecommunications, Nanjing 210023, China; Advanced Materials and Catalysis Group, Zhejiang Key Laboratory of Low-Carbon Synthesis of Value-Added Chemicals, State Key Laboratory of Clean Energy Utilization, Institute of Catalysis, Department of Chemistry, Zhejiang University, Hangzhou 310058, China; State Key Laboratory of Chemical Resource Engineering, Beijing Advanced Innovation Center for Soft Matter Science and Engineering, Beijing University of Chemical Technology, Beijing 100029, China; State Key Laboratory of Flexible Electronics (LoFE) & Institute of Advanced Materials (IAM), Nanjing University of Posts & Telecommunications, Nanjing 210023, China; State Key Laboratory of Flexible Electronics (LoFE) & Institute of Advanced Materials (IAM), Nanjing University of Posts & Telecommunications, Nanjing 210023, China; Institute of Functional Nano & Soft Materials (FUNSOM), Jiangsu Key Laboratory for Carbon-Based Functional Materials & Devices, Jiangsu Key Laboratory of Advanced Negative Carbon Technologies, Soochow University, Suzhou 215123, China; State Key Laboratory of Chemical Resource Engineering, Beijing Advanced Innovation Center for Soft Matter Science and Engineering, Beijing University of Chemical Technology, Beijing 100029, China; State Key Laboratory of Flexible Electronics (LoFE) & Institute of Advanced Materials (IAM), Nanjing University of Posts & Telecommunications, Nanjing 210023, China; Advanced Materials and Catalysis Group, Zhejiang Key Laboratory of Low-Carbon Synthesis of Value-Added Chemicals, State Key Laboratory of Clean Energy Utilization, Institute of Catalysis, Department of Chemistry, Zhejiang University, Hangzhou 310058, China

**Keywords:** polyoxometalates, anisotropic structures, emulsion assemblies, functional responsiveness, nanomotors

## Abstract

Polydopamine (PDA) is a versatile material known for its biocompatibility and adhesive properties, yet its intrinsic chemical inertness limits broader functional applications. Here, we report a one-pot polyoxometalate (POM)-mediated emulsion strategy for synthesizing hybrid mesoporous PDA (HMP) nanostructures with controllable anisotropic morphology and integrated functionality. Silicotungstic acid (H_4_SiW) directs the formation of P123/1,3,5-trimethylbenzene (TMB)/dopamine composite micelles, simultaneously stabilizing the emulsion, modulating interfacial tension, and templating hierarchical structures. This assembly pathway enables programmable shape evolution from isotropic nanospheres to open-cavity or bowl-shaped architectures. The embedded POMs remain molecularly dispersed and chemically active within the PDA framework, which directly endows the material with enhanced antibacterial functionality. Beyond structural sophistication, these HMPs can be driven by chemical reactions, enabling their use as artificial nanomotors. As a proof-of-concept application, Pt-loaded HMPs demonstrate self-propelled motion under H_2_O_2_ fuel and effective penetration and disruption of drug-resistant bacterial biofilms. Daptomycin-loaded nanomotors achieve near-complete eradication of drug-resistant *Staphylococcus aureus* biofilms. This work establishes a generalizable interfacial co-assembly approach for transforming inert PDA into multifunctional nanodevices, advancing the development of intelligent materials for biomedical and catalytic applications.

## Teaser

A one-pot POM-directed interfacial assembly strategy creates anisotropic mesoporous polydopamine nanomotors with intrinsic antibacterial activity, enabling Pt-driven propulsion, biofilm penetration, and near-complete eradication of drug-resistant Staphylococcus aureus biofilms.

## INTRODUCTION

Polydopamine (PDA), a biomimetic polymer inspired by the adhesive proteins of mussels, is synthesized via the oxidative polymerization of dopamine under alkaline conditions [1,2]. Owing to its strong adhesion, antioxidant properties, and excellent biocompatibility, PDA has found widespread applications in biosensing, bioimaging, drug delivery, and disease treatment [3–5]. However, despite these advances, pristine PDA exhibits limited stimuli-responsive behavior—a critical feature for developing adaptive and intelligent nanomaterials [6–9]. To address this limitation, various strategies have been explored to enhance the functional responsiveness of PDA, including copolymerization with functional monomers, post-synthetic surface modifications, and hybridization with other materials [10–15]. Nonetheless, achieving simultaneous control over both morphology and functionality—particularly the transition from isotropic to anisotropic structures—remains a significant challenge. Precise morphological tuning is essential, as anisotropic nanostructures often exhibit directional properties, compartmentalization, and enhanced responsiveness that are advantageous in fields such as catalysis, nanomotors, and targeted delivery [16–21]. Soft-templating approaches have been widely employed to fabricate mesoporous PDA nanostructures due to their versatility and simplicity [22–24]. A notable advancement introduced a co-assembly system involving Pluronic F127, 1,3,5-trimethylbenzene (TMB), and PDA to form

composite micelles at oil–water interfaces, paving the way toward anisotropic mesoporous architectures [25,26]. However, strategies enabling simultaneous *in situ* functional integration and precise shape control via multi-component co-assembly remain largely underexplored. This gap limits the development of functionally sophisticated PDA-based smart systems.

Polyoxometalates (POMs), a class of discrete, oxygen-rich metal-oxide clusters, present an attractive opportunity for addressing these challenges [27–29]. Their structural tunability, Brønsted acidity, redox activity, and ability to engage in diverse non-covalent interactions allow POMs to direct self-assembly processes and impart functionality. Importantly, their interactions with surfactants and polymers make them ideal candidates for modulating emulsion interfaces and promoting hierarchical structure formation [30–35].

Herein, we present a POM-mediated, emulsion-guided strategy for the synthesis of hybrid mesoporous PDA (HMP) nanostructures with controllable anisotropy and integrated functionality. The introduction of silicotungstic acid (H_4_SiW) serves as a key design element, enabling (i) stabilization of composite micelles comprising Pluronic P123, TMB, dopamine, and POMs, (ii) interfacial tension modulation to generate vesicle-like templates, and (iii) directed co-assembly into anisotropic morphologies. The resulting nanostructures exhibit tunable shape transitions, from isotropic spheres to anisotropic bowl-like architectures with hierarchical mesopores. Beyond structural sophistication, these HMPs exhibit autonomous motion under chemical gradients, enabling their use as artificial nanomotors. As a proof-of-concept application, Pt-loaded HMPs demonstrate directed chemotactic propulsion and effective penetration and disruption of drug-resistant bacterial biofilms. This study offers a generalizable interface engineering platform for the rational design of multifunctional PDA-based nanostructures, providing new opportunities in the development of intelligent nanodevices for biomedical and other advanced applications.

## RESULTS AND DISCUSSION

### Synthesis and characterization of HMP nanostructures

**Figure 1.**
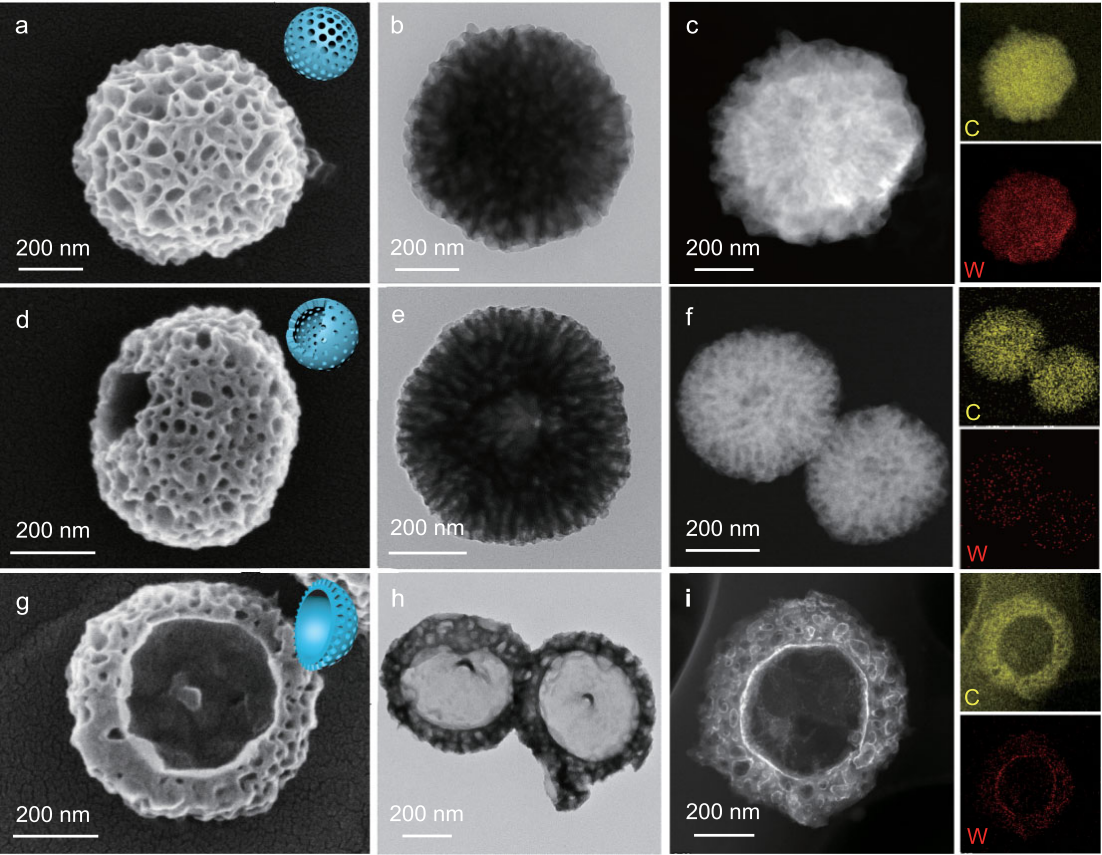
Controllable synthesis of HMPs with tunable morphologies. (a–c) SEM, TEM, and STEM images and corresponding EDX elemental mapping of representative HMP-0.4. (d–f) SEM, TEM, and STEM images with EDX mapping of HMP-1.0. (g–i) SEM, TEM, and STEM images with EDX mapping of HMP-1.5. Insets in panels (a), (d), and (g) show schematic models of the corresponding nanostructures with varying degrees of anisotropy.

The HMPs were synthesized via an emulsion-guided assembly process, using TMB as both a pore-expanding agent and oil phase, P123 as the surfactant and mesostructure-directing agent, and a combination of H_4_SiW and DA as functional components in an ethanol/water mixture. The incorporation of H_4_SiW facilitated the formation of stable P123/H_4_SiW/TMB/DA composite micelles, which underwent ordered co-assembly during dopamine polymerization. By varying the amount of TMB, we obtained a series of hybrid mesoporous nanoparticles with diverse morphologies, denoted as HMP-*x* (where *x* represents the TMB volume). Scanning electron microscopy (SEM) and transmission electron microscopy (TEM) analyses revealed the morphological evolution. At 0.4 mL of TMB (HMP-0.4), uniform spherical mesoporous particles (∼500 nm) were observed at different scales (Fig. 1a–c and Fig. S1). Increasing the TMB volume to 1.0 mL induced anisotropic growth, producing hollow spheres (∼600 nm) with distinct 1/8 open-mouth structures (Fig. 1d). High-resolution TEM images showed radially arranged mesopores (20–50 nm) and wall thicknesses of ∼25 nm (Fig. 1e). The energy-dispersive X-ray (EDX) elemental mapping confirmed uniform C and W distribution (Fig. 1f), and high-angle annular dark-field scanning transmission electron microscopy (HAADF-STEM) images indicated well-dispersed SiW_12_ units within the PDA matrix (Fig. S2). For HMP-1.5, SEM images (Fig. 1g) showed bowl-like particles (∼600 nm), and TEM (Fig. 1h) revealed large peripheral mesopores. Atomically resolved HAADF-STEM imaging (Fig. S3) further verified the molecular dispersion of SiW_12_ units. EDX mapping confirmed bowl-shaped frameworks with homogeneously distributed C and W (Fig. 1i).

**Figure 2.**
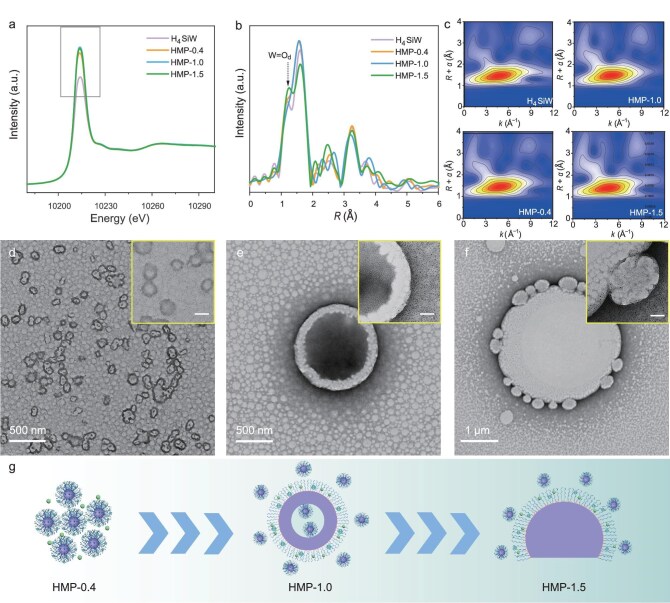
Fine structure characterization and evidence supporting the self-assembly mechanism. (a) Normalized W L_3_ edge XANES spectra, (b) FT-EXAFS spectra, and (c) Morlet wavelet transforms of HMP-0.4, HMP-1.0, HMP-1.5, and pristine H_4_SiW. TEM images of the emulsion systems prior to the addition of ammonium hydroxide: (d) HMP-0.4; (e) HMP-1.0; and (f) HMP-1.5. Insets in panels (d–f) show the enlarged images, with a scale bar of 100 nm. (g) Schematic illustration of emulsion morphologies corresponding to HMP-0.4, HMP-1.0, and HMP-1.5.

Thermogravimetric analysis revealed similar weight losses (∼91%) for HMP-0.4, HMP-1.0, and HMP-1.5 at 800°C (Fig. S4), with residual X-ray diffraction (XRD) peaks matching WO_3_ (JCPDS No. 20-1324), corresponding to ∼8.5%–9.8% of H_4_SiW content. The overall particle morphology remained well-preserved after calcination at 400°C (Fig. S5), confirming the excellent thermal stability of the hybrid framework materials (denoted as the HMC series). Nitrogen adsorption–desorption isotherms of HMC-1.0 and HMC-1.5 displayed type IV characteristics with pronounced hysteresis loops (0.8 < *P*/*P*_0_ < 1), indicative of large mesopores (Fig. S6). Barrett–Joyner–Halenda analysis showed broad pore distributions (25–60 nm), aligning with SEM/TEM observations. BET surface areas were 152.8 and 124.3 m^2^ g^−1^, respectively. XRD of the as-prepared particles showed a broad peak at ∼24.3°, corresponding to the (002) plane of amorphous carbon (Fig. S7) [36], with no crystalline H_4_SiW peaks, confirming molecular dispersion. Raman spectra showed characteristic D (∼1350 cm^−1^) and G (∼1590 cm^−1^) bands [37], confirming the coexistence of disordered sp^3^ and graphitic sp^2^ carbon in the PDA framework (Fig. S8a). Fourier-transform infrared spectra (Fig. S8b) confirmed the Keggin structure of SiW units via bands at 972, 925, 882, and 804 cm^−1^ [34], with slight shifts indicating strong interactions with PDA. X-ray photoelectron spectroscopy (Fig. S9) further confirms POM encapsulation through the distinct W and Si signals. X-ray absorption near-edge structure (XANES) spectra (Fig. 2a) revealed increased absorption intensities in the gray-boxed region for HMPs, likely due to enhanced W 5d orbital unoccupancy or distortion of the WO_6_ environment, suggesting pronounced electronic interactions between PDA and H_4_SiW during assembly. Extended X-ray absorption fine structure (EXAFS) spectra (Fig. 2b) demonstrated similar coordination environments to H_4_SiW with subtle shifts in W=O_d_ peaks, suggesting the interaction between H_4_SiW and the PDA support-induced distortions [38]. Wavelet transform patterns (Fig. 2c) further confirmed local coordination similarity, indicating successful incorporation of H_4_SiW without compromising its molecular integrity.

### Formation mechanism of nanohybrids with tunable morphologies

To understand the self-assembly mechanism, we examined the influence of DA dosage, catalyst amount, solvent ratio, and stirring rate using HMP-0.4 as a model. At low DA concentrations (0.05 g), irregular aggregates formed (Fig. S10). Increasing DA to 0.1 g yielded well-defined spheres with oriented mesochannels; further increases led to particle size growth (364 nm to 1.72 μm; Fig. S11). At 0.4 g of DA, secondary superstructure occurred. Varying ammonia concentration affected structural evolution (Fig. S12). Low ammonia (150–300 μL) induced phase separation; excess ammonia (600 μL) disrupted sphericity despite mesoporosity. Optimal conditions (∼450 μL, pH ≈ 10) preserved the Keggin structure of H_4_SiW, which otherwise degraded without PDA/P123 (Fig. S13). These results demonstrate that dopamine/P123/H_4_SiW co-assembly stabilizes POMs under weak alkaline conditions, primarily owing to the rapid polymerization kinetics of PDA, which promote prompt encapsulation of the POM clusters and thereby suppress their alkaline degradation (Fig. S14) [39]. Stirring rate also played a crucial role (Fig. S15): <800 r/min yielded irregular products; ≥800 r/min produced uniform spheres. At 1500 r/min, bowl-like architectures emerged, similar to those formed at higher TMB levels. This suggests stirring modulates emulsion stability, affecting micelle fusion and particle shape. Control experiments without H_4_SiW produced dendritic PDA spheres (∼185 nm, MP-0.4; Fig. S16), indicating that H_4_SiW is key for anisotropic growth. Substituting H_4_SiW with other acids (HCl, HNO_3_, H_2_SO_4_) at pH ≈ 2 yielded MP-0.4-like structures (Figs S17 and S18), confirming that acidity alone does not drive anisotropic morphology.

TMB, acting as both an oil phase and pore expander, played a critical role. Without TMB, irregular aggregates formed (Fig. S19); increasing TMB promoted pore formation and morphology transition from solid spheres (0.4 mL) to open-mouthed spheres (1.0 mL) and bowl-like structures (1.5 mL). In contrast, morphology remained unchanged in H_4_SiW-free systems regardless of TMB (Fig. S20), reinforcing the essential role of H_4_SiW. *Ex situ* TEM of intermediate emulsions before ammonia addition showed distinct structural transformations. At 0.4 mL of TMB, ∼70 nm spherical composite micelles formed (Fig. 2d). These micelles likely serve as nucleation sites for the subsequent co-assembly into the final nanospheres. At 1.0 mL, vesicle-like structures with H_4_SiW enrichment at interfaces appeared (Fig. 2e). At 1.5 mL, synapse-like protrusion structured emulsion formed (Fig. 2f), aligning with final bowl morphologies. Similarly, the presence of H_4_SiW dots at the edges of the protrusions reinforced their preferential interfacial localization. This observation is further supported by the significant reduction in interfacial tension caused by H_4_SiW (Fig. S21), which behaves like a surfactant in stabilizing the hierarchical emulsions. Its efficient non-covalent interactions with the PEO blocks of the nonionic surfactant F127, stabilized at the oil (TMB)–water interface [40,41], reduce the interfacial energy of the composite emulsions, thereby facilitating the formation of hierarchical emulsion structures. Control systems lacked such intermediates (Figs S22 and S23). Upon ammonia-triggered polymerization, isotropic PDA-based porous nanospheres (HMP-0.4) emerge from the co-assembly and polymerization of spherical composite micelles. As for 1.0 mL of TMB (HMP-1.0), in contrast, the deformation of meta-stable vesicles contributed to the hollow structures with 1/8 open apertures. This deformation is driven by the migration of PDA/oligomers into the emulsion phase, facilitated by the complete wetting of PDA on TMB (contact angle ∼0°, Fig. S24). This speculation accounts for the smaller diameter (∼600 nm) and cavity (∼200 nm) of HMP-1.0 relative to the vesicle (∼1 μm) and its interior (∼800 nm) (Fig. S25) [42]. HMP-1.5 are deemed to originate from the templated replication of bowl-like micelle protrusions. Figure 2g shows the schematic illustration of emulsion morphologies corresponding to HMP-0.4, HMP-1.0, and HMP-1.5.

**Figure 3.**
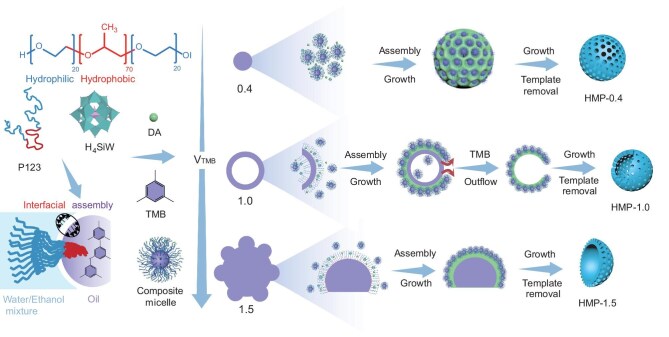
Proposed formation mechanism of HMPs. Schematic illustration of the possible formation pathway of HMP nanostructures in emulsion systems with varying volumes of TMB.

Overall, a POM-mediated emulsion assembly mechanism was identified (Fig. 3). Under stirring, co-assembly of TMB, P123, H_4_SiW, and DA formed composite micelles. Varying TMB modulated emulsion morphology, leading to distinct nanostructures—from isotropic spheres to hierarchical bowls—by tuning interfacial interactions and polymerization dynamics. This strategy is generalizable to other solvents (Fig. S26) and mixed surfactants (Fig. S27), highlighting the considerable potential of this POM-mediated emulsion strategy (Figs S28–S31).

### Motion analysis of the HMPM nanomotors

**Figure 4.**
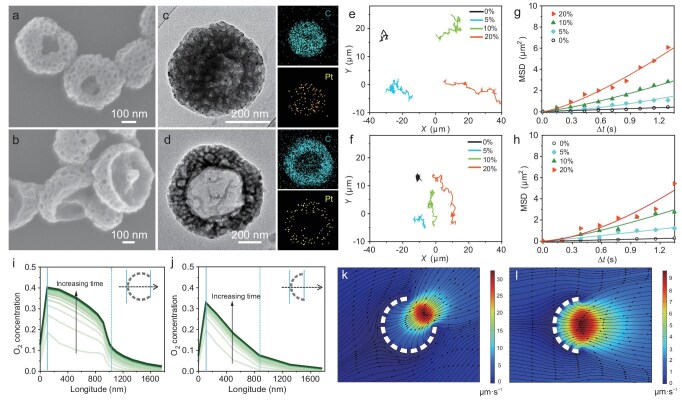
Self-propelled behavior of HMPMs in hydrogen peroxide solutions. (a and b) SEM images, (c and d) HRTEM images and corresponding EDX elemental mappings of (a and c) HMPM-1.0 and (b and d) HMPM-1.5. (e and f) Representative 10 s trajectories and (g and h) MSD curves of (e and g) HMPM-1.0 and (f and h) HMPM-1.5 in hydrogen peroxide solutions with varying concentrations. (i and j) Simulated O_2_ concentration profiles along the symmetry axis of (i) HMPM-1.0 and (j) HMPM-1.5. (k and l) Simulated fluid velocity distribution (black-arrow) around (k) HMPM-1.0 and (l) HMPM-1.5 at 1 μs.

Anisotropic particle geometry is widely recognized as a key design principle for enabling directional self-propulsion in micro/nanomotors [43–47]. Here, we incorporated platinum nanoparticles (Pt NPs) into HMPs to construct colloidal nanomotors (HMPM series) with tunable degrees of anisotropy (Fig. 4a–d and Fig. S32). The Pt NPs catalyze the decomposition of hydrogen peroxide (H_2_O_2_), generating oxygen to drive motion via diffusiophoresis [48–51]. Upon dispersal in H_2_O_2_ solutions, the motility of HMPMs was evaluated via optical microscopy. In the absence of H_2_O_2_, all nanomotors displayed nondirectional Brownian motion. With increasing H_2_O_2_ concentration, motor displacement progressively increased, indicating active propulsion. Representative trajectories of HMPM-1.0 and HMPM-1.5 at varying H_2_O_2_ levels are shown in Fig. 4e and f. Notably, HMPM-0.4 retained random motion even at 20% H_2_O_2_, underscoring the essential role of geometric asymmetry (Figs S33 and S34). Mean square displacement (MSD) versus time interval (Δ*t*) plots (Fig. 4g and h) demonstrated increasing slope with higher fuel concentration, reflecting a transition from Brownian diffusion to active motion. The calculated effective diffusion coefficients (*D*_eff_ = MSD/4Δ*t*) confirmed enhanced motility in a fuel-dependent manner (Fig. S35). Importantly, HMPM-1.0 exhibited faster propulsion than HMPM-1.5, attributed to its smaller 1/8 aperture that promotes greater confinement of O_2_ and thus stronger propulsion force. Finite element simulations (Fig. 4i–l and Fig. S36) revealed asymmetric concentration gradients within the nanomotor cavities. O_2_ concentration was elevated while H_2_O_2_ was depleted inside the cavities, generating directional chemical gradients to drive motion. The steeper gradient in HMPM-1.0 compared to HMPM-1.5 corroborates the experimental observation of its faster speed.

### Biofilm penetration and destruction of HMPM nanomotors

**Figure 5.**
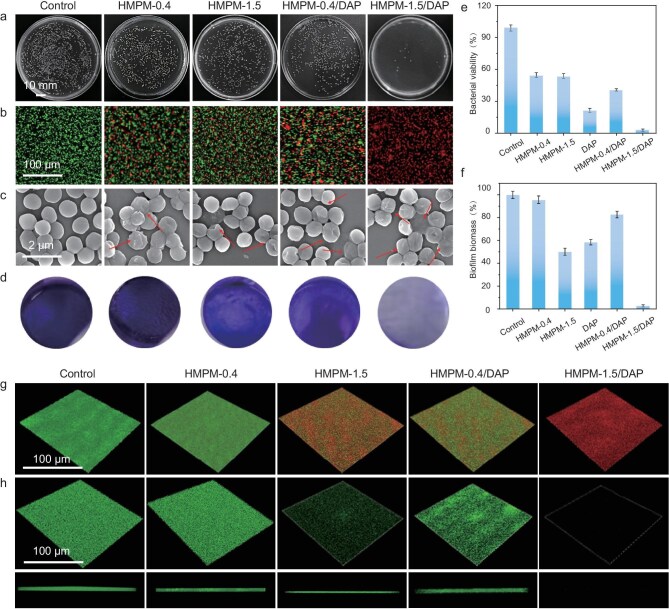
Antibacterial activity of HMPM-1.5 and HMPM-1.5/DAP *in vitro*. (a) Photographs of colonies after different treatments. (b) Live/dead staining images of MRSA after different treatments. (c) SEM images of MRSA after different treatments (the collapsed bacterial skeletal structure clearly marked by red arrows). (d) Crystal violet staining of MRSA after different treatments. (e) Bacterial viability of MRSA after different treatments: control, HMPM-0.4, HMPM-1.5, DAP, HMPM-0.4/DAP, and HMPM-1.5/DAP. (f) Biofilm biomass at OD_595_ using ethanol. (g) 3D views of live/dead staining images of the biofilms after different treatments. (h) 3D views of SYTO 9 staining images of the biofilms after different treatments.

Bacterial infection remains a major barrier to effective chronic wound healing. As shown in Fig. S37, the antibacterial efficiencies of HMP-1.5 and HMP-0.4 are 43.6% and 40.2% respectively. In contrast, pure PDA nanoparticles—both isotropic PDA (I-PDA) and anisotropic PDA (A-PDA)—showed only ∼7% antibacterial efficiency. These results indicate that the *in situ* encapsulation of POMs is responsible for endowing the PDA matrix with pronounced antibacterial activity [52,53]. To further assess the drug-loading capacity of the nanomotors, daptomycin (DAP) was loaded into both HMPM-0.4 and HMPM-1.5, and the ultraviolet-visible (UV-vis) absorbance at 370 nm was monitored. As shown in Fig. S38, the characteristic absorption peak of DAP at 370 nm was clearly observed in both HMPM-0.4-DAP and HMPM-1.5-DAP samples, confirming successful drug loading. Quantitative analysis revealed that at a mass ratio of DAP:HMPM = 10:1, the loading content (LC) and encapsulation efficiency (EE) of HMPM-1.5 reached 930% and 93.0%, respectively (Figs S39 and S40). In contrast, HMPM-0.4 exhibited significantly lower values, with an LC of 110% and an EE of 11.0%. The superior drug-loading performance of HMPM-1.5 is attributed to its unique anisotropic cavity structure, which maximizes drug encapsulation capacity. Beyond drug loading, the anisotropic architecture of HMPM-1.5 imparts efficient self-propulsion, enabling autonomous motion in response to infection-associated microenvironments with locally elevated H_2_O_2_ concentrations [54,55]. This active locomotion enhances bacterial targeting and biofilm penetration while minimizing damage to surrounding healthy tissues [56–58]. Such properties underscore the promise of HMPM-1.5 as an advanced nanomotor platform for the treatment of biofilm-associated bacterial infections. Moreover, the biocompatibility of HMPM-1.5 and DAP-loaded HMPM-1.5 (HMPM-1.5-DAP) was systematically assessed. Cytotoxicity against NIH-3T3 fibroblasts was evaluated using the MTT assay. As shown in Fig. S41a, both HMPM-1.5 and HMPM-1.5-DAP exhibited negligible cytotoxicity at concentrations up to 150 μg mL^−1^, indicating excellent biocompatibility. Hemolysis assays using red blood cells confirmed the excellent biocompatibility of the materials, with hemolysis rates of HMPM-1.5 and HMPM-1.5/DAP remaining below the standard value of 5% even at concentrations up to 200 μg mL⁻¹ (Fig. S42), indicating negligible hemolytic effects. The antibacterial activity of HMPM-1.5 and HMPM-1.5-DAP against methicillin-resistant *Staphylococcus aureus* (MRSA) was then evaluated using the spread plate method [59,60]. HMPM-1.5-DAP demonstrated significantly enhanced antibacterial activity compared to pristine HMPM-1.5, with a clear concentration-dependent effect observed from bacterial colony counts (Fig. S41b). DAP alone showed a strong antibacterial effect, achieving 78.5% bacterial inhibition (Fig. 5e and Fig. S43a). Notably, HMPM-1.5-DAP achieved an exceptional antibacterial efficiency of 97.8%, outperforming HMPM-0.4-DAP (59.3%) (Fig. 5a and e). This enhancement can be attributed to the higher DAP loading capacity of HMPM-1.5 and its anisotropic structure, which facilitates autonomous propulsion, enhances bacterial targeting, and promotes efficient drug release at infection sites. These results collectively demonstrate that the self-propelling capability of HMPM-1.5 not only improves bacterial targeting via increased collision frequency and prolonged surface retention, but also enables efficient and controlled DAP delivery through an active transport mechanism. SYTO 9/PI staining was used to visualize bacterial viability after treatment. Dead and live bacteria were differentiated by the fluorescence color (red fluorescent spots were dead bacteria, green spots were live bacteria) (Fig. 5b and Fig. S43b). The bacteria treated with HMPM (HMPM-0.4 and HMPM-1.5), DAP or HMPM-0.4-DAP showed a certain degree of death, which was consistent with the results in Fig. 5a and e. Among them, no viable bacteria were identified in the HMPM-1.5-DAP group, indicating its optimal antibacterial effect. Moreover, SEM analysis in Fig. 5c and Fig. S43c showed that HMPM-1.5-DAP treatment significantly induced bacterial surface perforation and leakage of cellular contents, which confirmed that HMPM-1.5-DAP could cause the collapse of bacterial skeleton structure.

The outstanding catalytic performance and fast movement of HMPM-1.5 highlighted its strong potential in combating biofilms. Consequently, we examined the ability of HMPM-1.5 to infiltrate MRSA biofilm structures. Intact biofilms stained with SYTO 9 were co-incubated with rhodamine B-tagged HMPM-1.5 for fluorescence visualization. The penetration depth variations in the biofilm structure were evaluated using 3D confocal laser scanning microscope (CLSM) by monitoring the red fluorescence signal. In Fig. S44, a widespread fluorescence distribution was detected across the biofilm matrix, demonstrating successful infiltration of HMPM-1.5 in biofilm. Based on this, we further evaluated the removal efficiency of different treatments on bacterial biofilms via crystal violet staining assays. In Fig. 5d and f, HMPM-0.4 exhibited merely 4.3% of MRSA biofilm eradication due to its erratic movement, whereas HMPM-1.5 achieved 49.9% of biofilm clearance by virtue of its superior penetration capacity, which was comparable to DAP alone (41.6%; Fig. 5f and Fig. S43d). The HMPM-1.5-DAP combination demonstrated dramatically enhanced biofilm elimination (97.2%) over HMPM-0.4-DAP (17.2%), as the autonomous motility of HMPM-1.5 and rapid DAP release synergistically boosted therapeutic efficacy. Subsequently, the viability of bacteria in the biofilms was investigated by SYTO 9/PI staining (Fig. 5g and Fig. S45a). Consistent with the crystal violet staining results, HMPM-1.5 demonstrated superior bactericidal efficacy against biofilm-embedded bacteria compared with HMPM-0.4 alone, and nearly all bacteria were eradicated with the DAP-loaded HMPM-1.5, demonstrating the efficient permeation and distribution throughout the biofilm matrix, enabling drug release in deeper regions to eliminate pathogens. Furthermore, we conducted SYTO 9 staining to assess the biofilm thickness following various treatments. In Fig. 5h and Fig. S45b, the biofilm thickness remained nearly unchanged after treatment with HMPM-0.4 alone, while only minor alterations were observed after treatment with HMPM-0.4-DAP. In contrast, both HMPM-1.5 and DAP significantly reduced biofilm thickness, with near-complete biofilm eradication achieved in the HMPM-1.5-DAP combination group. These findings demonstrated that our motor-driven system could successfully destroy biofilms and kill bacteria via POM-enabled functionality and anisotropy-enabled movement.

## CONCLUSION

In summary, we developed a POM-mediated emulsion-directed strategy for constructing anisotropic, functionalized mesoporous PDA nanostructures. By leveraging the multifaceted roles of silicotungstic acid in stabilizing micelles and regulating interfacial assembly, we achieved precise control over both particle shape and internal functional composition. The resulting anisotropic architectures enable self-propulsion under chemical gradients, enhanced drug loading, and biofilm penetration. Pt-loaded nanomotors exhibit effective chemotactic behavior and strong antibacterial performance against drug-resistant biofilms. This work demonstrates a versatile route for integrating shape anisotropy and active functionality into PDA nanostructures, overcoming intrinsic material limitations and offering new possibilities for the design of intelligent nanodevices in biomedical and other high-performance applications.

## METHOD AND MATERIALS

### Synthesis of hybrid mesoporous polydopamine particles (HMP-*x*)

Typically, 40 mg of tungstosilicic acid hydrate (H_4_SiW, Aladdin) was dissolved in 5 mL of water, followed by the addition of 0.1 g of P123 (PEO_20_PPO_70_PEO_20_, *M*w = 5800 g mol^−1^, Sigma-Aldrich) under stirring for 3 h to obtain a clear solution. Subsequently, 1 mL of water, 3 mL of ethanol, *x* mL of TMB (Aladdin), and 0.15 g of dopamine hydrochloride (DA, Sigma–Aldrich) were sequentially added to form a homogeneous nanoemulsion. After 30 min of equilibration, 0.45 mL of concentrated ammonia was introduced to initiate DA self-polymerization. The reaction proceeded for 6 h at 20°C in a water bath, yielding hybrid mesostructured H₄SiW/P123/TMB/PDA assemblies (denoted as HMP-*x*), which were collected by centrifugation, washed thoroughly with water and ethanol, and dried at 60°C overnight. Corresponding carbon materials (denoted as HMC-*x*) could be easily produced after pyrolysis of the polymerized HMP-*x* by calcination at 400°C for 3 h under N_2_ atmosphere with a heating rate of 1°C min^−1^. When ethanol was replaced by tetrahydrofuran, 1,4-dioxane, and acetone as the co-solvent, the resulting products were labeled as HMP-THF-*x*, HMP-DIO-*x*, and HMP-ACE-*x*, respectively.

### Synthesis of mesoporous polydopamine particles (MP-*x*)

The synthesis procedure was identical to that of HMP-*x*, except that H_4_SiW was omitted from the initial solution.

Synthesis of PDA used for contact angle test: Typically, 0.3 g of dopamine hydrochloride was dissolved in 20 mL deionized water, followed by the addition of 0.9 mL concentrated ammonia to initiate polymerization. After stirring for 6 h at room temperature, the resulting product was collected by centrifugation, washed sequentially with deionized water and ethanol three times, and dried at 60°C overnight.

Fabrication of colloidal motors: Specifically, 10 mg of HMP-*x* (*x* = 0.4, 1.0, 1.5) was ultrasonically dispersed in 5 mL of deionized water. Then, 0.3 mL of aqueous H_2_PtCl_6_ solution (10 mg mL^−1^) was added and stirred for 30 min. Subsequently, 1 mL of aqueous NaBH₄ solution (2.77 mg mL^−1^) was introduced to reduce the Pt precursor. After 10 min of reaction, the products were collected by centrifugation, washed with water, and denoted as HMPM-*x*, corresponding to isotropic and anisotropic colloidal motors with different morphologies.

## Supplementary Material

nwag214_Supplemental_File
