# Supplementary material for: Polyoxometalate-directed interfacial assembly of multifunctional mesoporous polydopamine nanomotors
Source: Natl Sci Rev. 2026 Apr 7;13(10):nwag214. doi: 10.1093/nsr/nwag214 (PMC13221958; doi:10.1093/nsr/nwag214)
Supplement: nwag214_Supplemental_File [file nwag214_supplemental_file.pdf]

Polyoxometalate-directed interfacial assembly of multifunctional mesoporous polydopamine nanomotors

Chunhong Chen[1,†], Lihua Wang[2,†], Yu Yang[3,†], Tengjin Wang[1], Ting Li[1], Hao Zhang[4], Ruizheng Liang[3,*], Lianhui Wang[1,*], and Yong Wang[2,*]

[1]State Key Laboratory of Flexible Electronics (LoFE) & Institute of Advanced Materials (IAM), Nanjing University of Posts & Telecommunications, Nanjing 210023, China;

[2]Advanced Materials and Catalysis Group, Zhejiang Key Laboratory of Low-Carbon Synthesis of Value-Added Chemicals, State Key Laboratory of Clean Energy Utilization, Institute of Catalysis, Department of Chemistry, Zhejiang University, Hangzhou 310058, China;

[3]State Key Laboratory of Chemical Resource Engineering, Beijing Advanced Innovation Center for Soft Matter Science and Engineering, Beijing University of Chemical Technology, Beijing 100029, China;

[4]Institute of Functional Nano & Soft Materials (FUNSOM), Jiangsu Key Laboratory for Carbon-Based Functional Materials & Devices, Jiangsu Key Laboratory of Advanced Negative Carbon Technologies, Soochow University, Suzhou 215123, China

*Corresponding authors. E-mails: chemwy@zju.edu.cn; iamlhwang@njupt.edu.cn; liangrz@buct.edu.cn

[†]Equally contributed to this work.

Experimental Section

Optical microscopy observation

The motion behavior of HMPM-x was observed using an Olympus CKX41 inverted microscope. H_2O_2 solutions of varying concentrations were first placed onto a hydrophilic glass slide, followed by the addition of a diluted HMPM-x suspension. After allowing the system to stabilize for several minutes, 10-second videos were recorded using a CCD camera.

Movement analysis: Tracking of the nanomotors was performed using ImageJ software [1]. The mean square displacement (MSD) was calculated according to the following equation:

$$\mathrm{MSD}(\Delta t) = \langle [x(t+\Delta t) - x(t)]^2 + [y(t+\Delta t) - y(t))]^2 \rangle \quad (1)$$

where $x(t)$ and $y(t)$ represent the coordinates of the nanomotor at time t, and Δt is the time interval. The angle brackets $\langle\ \rangle$ denote ensemble averaging over time origins and particle trajectories. To minimize tracking errors, only the initial 5 seconds of motion data were used for MSD analysis. The translational diffusion coefficients (D_{eff}) were extracted by fitting the MSD curves with equation:

$$\mathrm{MSD} = 4D_{\mathrm{eff}}\Delta t \quad (2)$$

Preparation of HMPM/DAP: The pre-synthesized HMPM was firstly dissolved in PBS, followed by the addition of daptomycin (DAP) with a mass ratio of HMPM: DAP=1:10, After stirring at room temperature for 12 h, the resulting HMPM/DAP was obtained by centrifugation at 6000 r $\min^{-1}$ for 3 times and re-dispersed in PBS for further use.

Determination of LC and EE: The concentration of DAP in the initial drug solution and supernatant was measured using a Shimadzu U-3000 spectrophotometer based on its absorbance at 370 nm. The LC and EE of DAP were calculated according to the following equations:

$$\mathrm{LC} = (\mathrm{W_{Fed}} - \mathrm{W_{non-encapsulated}})/\mathrm{W_{HMPM}} \times 100\% \quad (3)$$

$$EE = (W_{Fed} - W_{non-encapsulated})/W_{Fed} \times 100\% \quad (4)$$

W_{Fed} is the initial total mass of fed drug, $W_{non\text{-}encapsulated}$ is the drug mass in supernatant after centrifugation, and W_{HMPM} is the mass of HMPM added in the loading process.

Cytotoxicity evaluation: 3T3-Swiss albino (3T3) cells were colonized in 96-well plates and cultured overnight. Then, the cells were treated with different concentrations (0, 12.5, 25, 50, 75, 100, and 150 μg mL^{-1}) of HMPM-1.5 and HMPM-1.5/DAP for 24 h to investigate their biocompatibility. The medium was replaced with a refresh culture medium containing methylthiazolyldiphenyl-tetrazolium bromide (MTT, 200 μL per well, 500 μg mL^{-1}) and incubated at 37°C for 4 h. Finally, DMSO (200 μL per well) was added after discarding the previous medium, and the cell viability was measured with a microplate reader.

In vitro antibacterial assays

MRSA suspensions (10^7 CFU mL^{-1}) were co-incubated with 1 mL suspension of different groups (HMPM0.4, HMPM1.5, DAP, HMPM0.4/DAP, and HMPM1.5/DAP, 100 μg mL^{-1} of HMPM, 100 μg mL^{-1} of DAP) in PBS under acidic (pH=6.5) condition. The suspension (10 μL) was then diluted and inoculated on a prepared LB medium in a humidified incubator with a constant temperature of 37°C for 12 h. Bacterial growth and colony numbers on LB agar plates were photographed and counted. To access the antibacterial efficiency intuitively, bacterial pellets from different treatment groups were fixed with 2.5% glutaraldehyde (4°C, 12 h), followed by gradient ethanol dehydration (30%, 50%, 70%, 90%, 100% v/v). The morphology of bacteria was recorded by a SEM. For the live-dead staining assay, bacterial suspensions after different treatments were dual-stained with SYTO 9 and PI for 15 min in the dark. Fluorescence imaging was acquired using an inverted laboratory microscope.

Infiltration of HMPM1.5 into biofilms

The 48 h intact biofilms were exposed to aqueous dispersion of RhB-labeled HMPM1.5 (100 μg mL^{-1}) with different times (0, 2, 4, 6, 8, 10 min). Then, bacteria were stained

with SYTO 9 to visualize the biofilms and observed by 3D CLSM at different time points.

Antibiofilm properties

Biofilm inhibition capability was evaluated using a crystal violet quantitative assay. *MRSA* suspensions (10^8 CFU mL^{-1}) were seeded into 48-well plates and incubated at 37°C for 48 h to facilitate biofilm maturation. After that, the planktonic bacteria were thoroughly washed with PBS, followed by specific treatments for each group (HMPM0.4, HMPM1.5, DAP, HMPM0.4/DAP, and HMPM1.5/DAP, 100 μg mL^{-1} of HMPM, 100 μg mL^{-1} of DAP). Subsequently, air-dried biofilms were fixed with 200 μL methanol (10 min), followed by staining with 5% crystal violet solution for 30 min. After removing excess staining solution and treatment with ethanol, the absorbance of the biofilm at 590 nm was measured using microplate reader. For fluorescence staining analysis, slides were pre-inserted into the 6-well plates before biofilm incubation. After air-drying, the slides were coated with live/dead staining solution, and the samples were incubated in the dark for 30 min at 37°C. The final bacterial fluorescence staining images were observed using a confocal laser scanning microscope.

Characterization techniques

Low-magnification TEM images were collected by a Hitachi HT7700 at an acceleration voltage of 120 kV. Scanning electron microscope (SEM) measurements were conducted on a Hitachi SU-8010 electron microscope. High-resolution transmission electron microscopies (HRTEM) and Energy-dispersive X-ray (EDX) spectral analysis were conducted on JEOL JEM-2100F. High-angle annular dark-field scanning TEM (HAADF-STEM) was collected using a FEI Tian G2 80-200 ChemiSTEM at an operation voltage of 200 kV. Powder X-ray Diffraction (XRD) patterns were recorded on a Rigaku Ultima IV X-ray diffractometer operating at 40 kV and 30 mA (Cu Kα, $\lambda = 1.5418$ Å) over the range of $10° < 2\theta < 80°$ in 0.05° step width with a 10°/min scanning speed. The Brunauer-Emmett-Teller (BET) surface areas and porous structures of the samples were determined using a Micromeritics ASAP 2020 HD88.

The X-ray photoelectron spectra (XPS) were recorded on an Escalab 250Xi spectrometer. Fourier transform infrared (FT-IR) spectroscopy was performed using a Perkin-Elmer Spectrum One FTIR spectrometer with diamond ATR accessory using KBr pellets. TG analysis was performed on a TA SDT Q600 with heating rate of 10°C/min. The Raman spectra are collected on a Raman spectrometer (JY, HR 800) using a 514 nm laser. The water and oil interfacial tension and contact angle are measured by the contact angle surface tension meter of Dropmeter 100P. X-ray absorption fine structure (XAFS) measurements at W L_3 edge in transmission mode were performed at the BL14W1 in Shanghai Synchrotron Radiation Facility (SSRF). The electron beam energy was 3.5 GeV and the stored current was 230 mA (top-up). A 38-pole wiggler with a maximum magnetic field of 1.2 T inserted in the straight section of the storage ring was used. XAFS data were acquired using a fixed-exit double-crystal Si (111) monochromator. The raw data analysis was collected using IFEFFIT software package according to the standard data analysis procedures [2]. The spectra were calibrated, averaged, pre-edge background subtracted, and post-edge normalized using Athena program in IFEFFIT software package. The Fourier transformation of the k^2-weighted EXAFS oscillations, $k^2 \cdot \chi(k)$, from k space to R space was performed over a range of 2.6-12.3 $Å^{-1}$ to obtain a radial distribution function. UV-vis spectrum was recorded on a TU-1950 spectrophotometer.

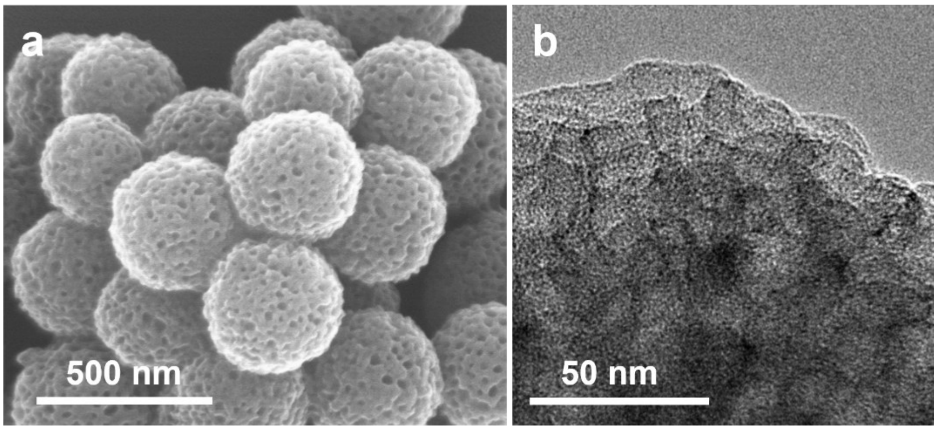

Figure S1. (a) SEM and (b) TEM images of HMP-0.4.

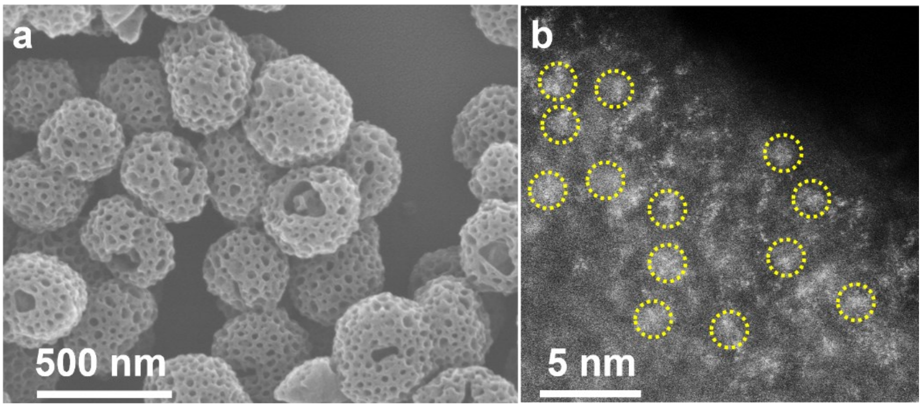

Figure S2. SEM (a) and aberration-corrected HAADF-STEM (b) image of HMP-1.0.

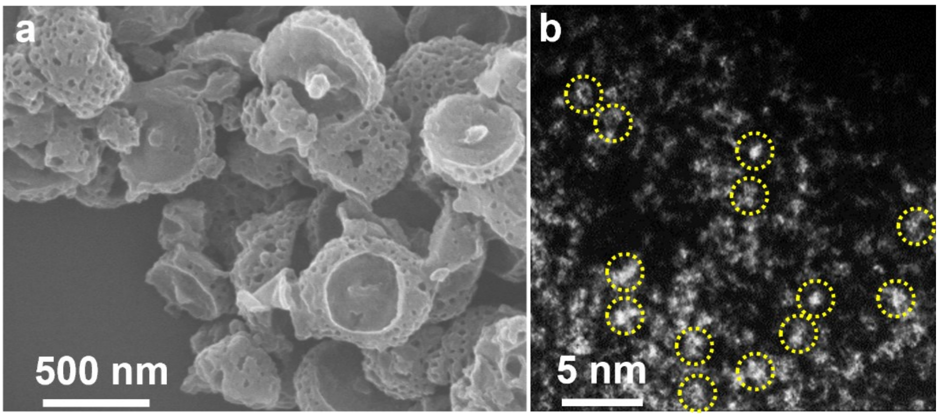

Figure S3. SEM (a) and aberration-corrected HAADF-STEM (b) image of HMP-1.5.

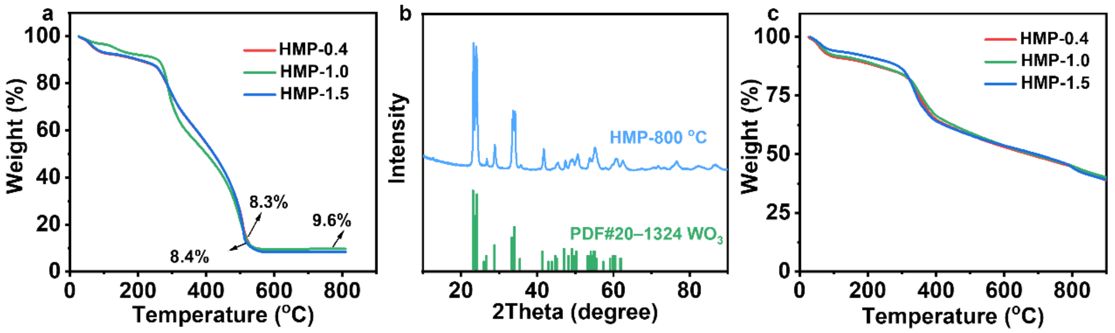

Figure S4. a,b, TG curves of HMP-x in air (a) and XRD patterns of the residue of HMP-0.4 after calcination at 800°C in air (b). c, TG curves of HMP-x in N_2.

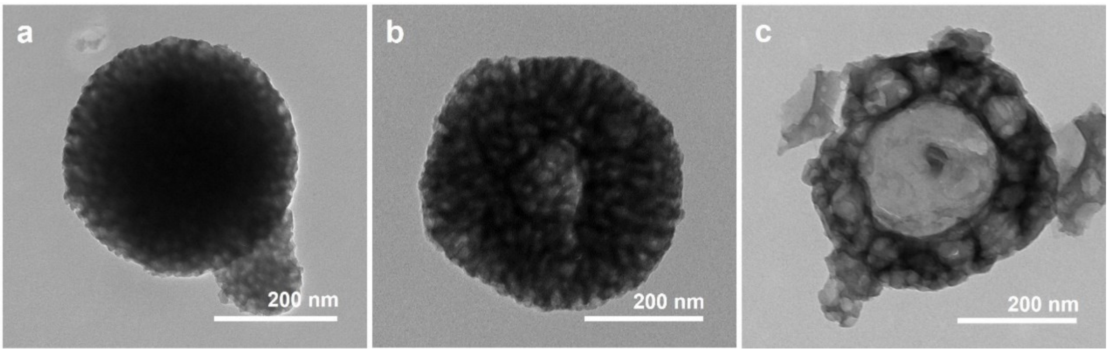

Figure S5. a–c, TEM images of HMC-0.4 (a), HMC-1.0 (b), and HMC-1.5 (c).

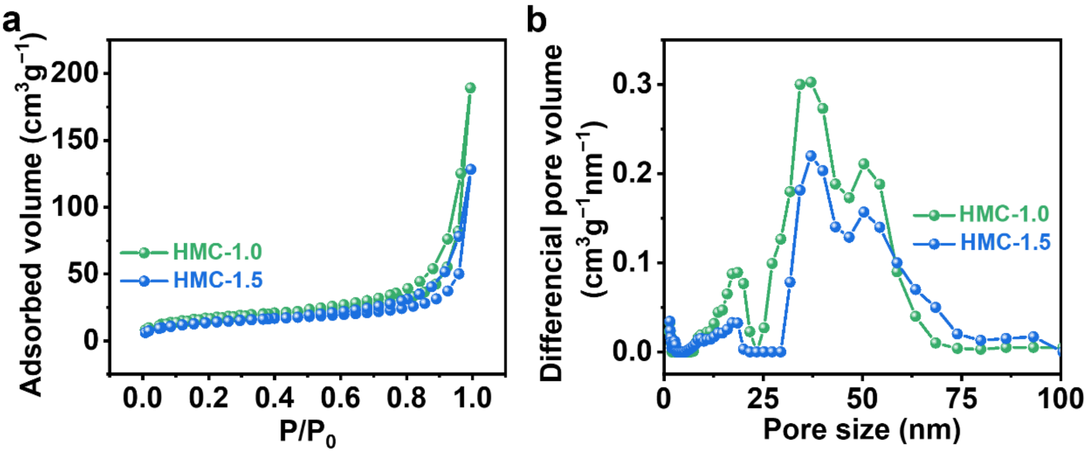

Figure S6. a,b, N_2 adsorption-desorption isotherms (a) and the corresponding BJH pore size distribution curves (b) of HMC-1.0 and HMC-1.5.

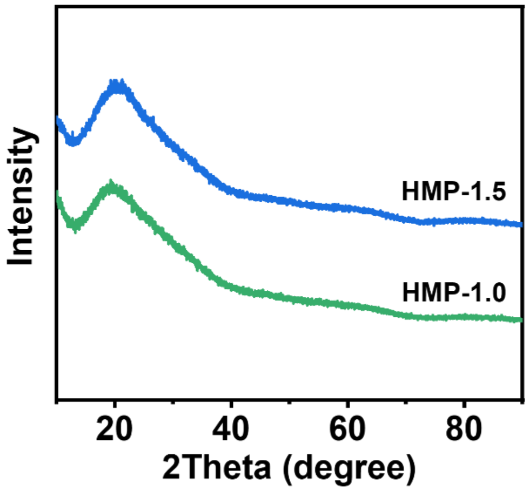

Figure S7. XRD patterns of HMP-1.0 and HMP-1.5.

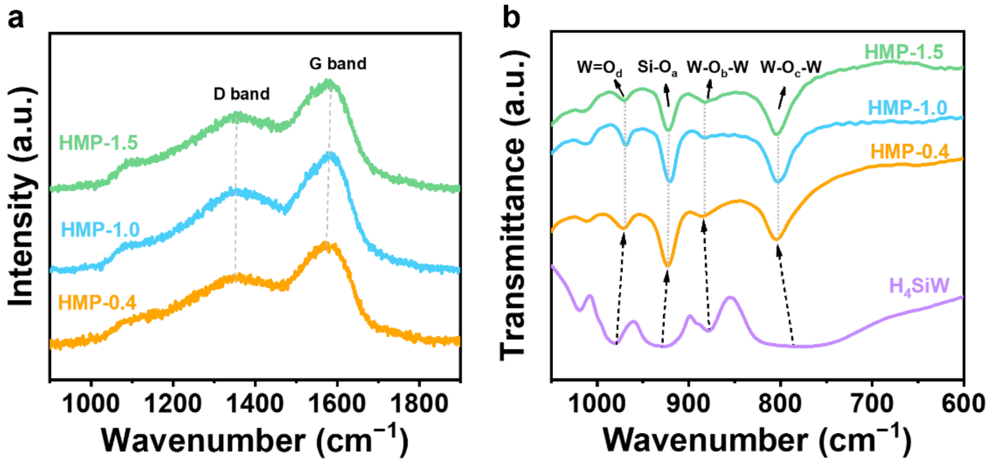

Figure S8. a,b, Raman spectra (a) and FT-IR spectra (b) of HMP-0.4, HMP-1.0, HMP-1.5, and pristine H_4SiW.

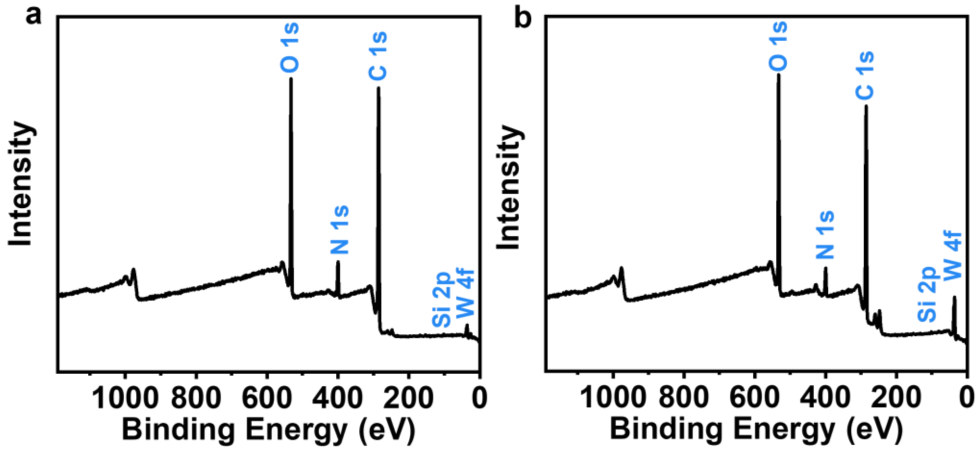

Figure S9. a,b, XPS full survey of (a) HMP-1.0 (a) and HMP-1.5 (b).

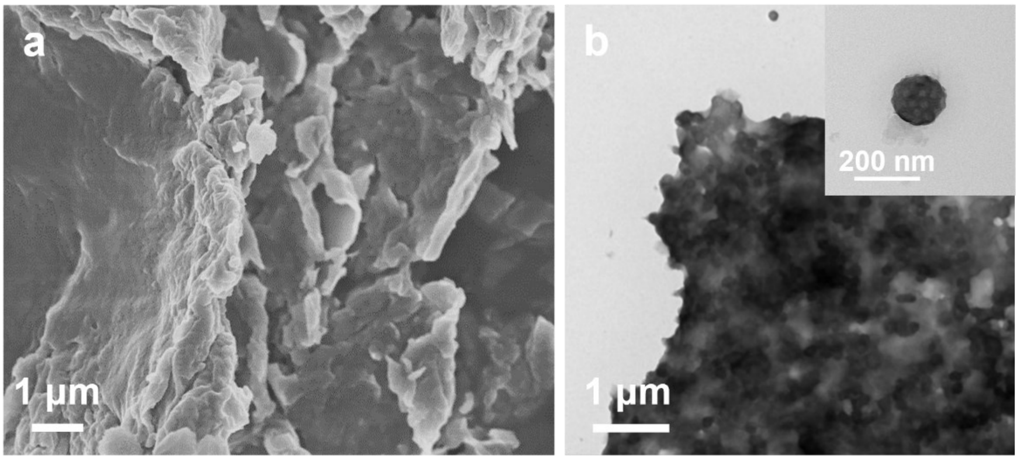

Figure S10. a,b, SEM (a) and TEM (b) images of HMP-0.4 when the dosage of DA is 0.05 g.

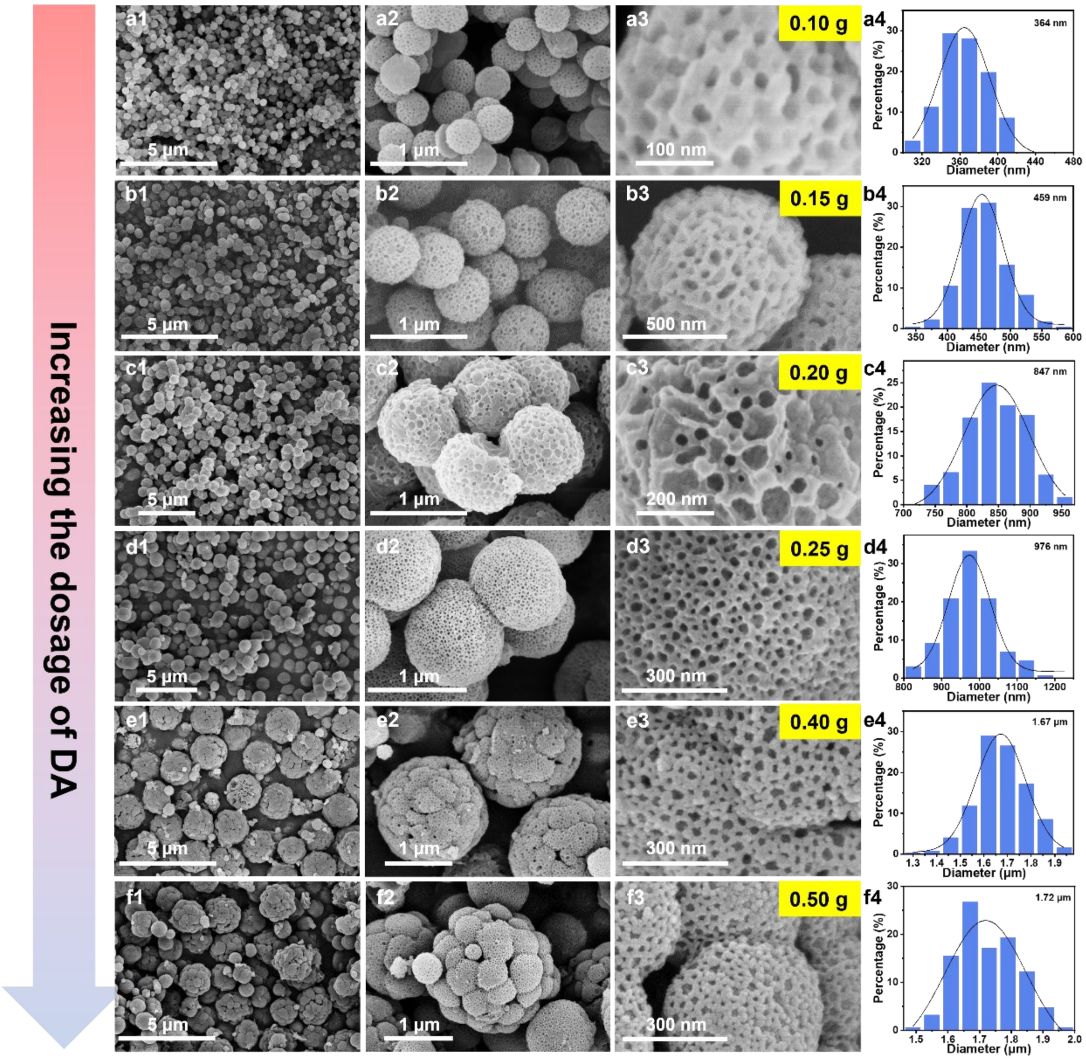

Figure S11. a–f, SEM images and corresponding particle size distribution histograms (calculated from over 100 particles) of the prepared mesoporous HMP-0.4 with different amounts of DA: 0.10 g (a), 0.15 g (b), 0.20 g (c), 0.25 g (d), 0.40 g (e), and 0.50 g (f).

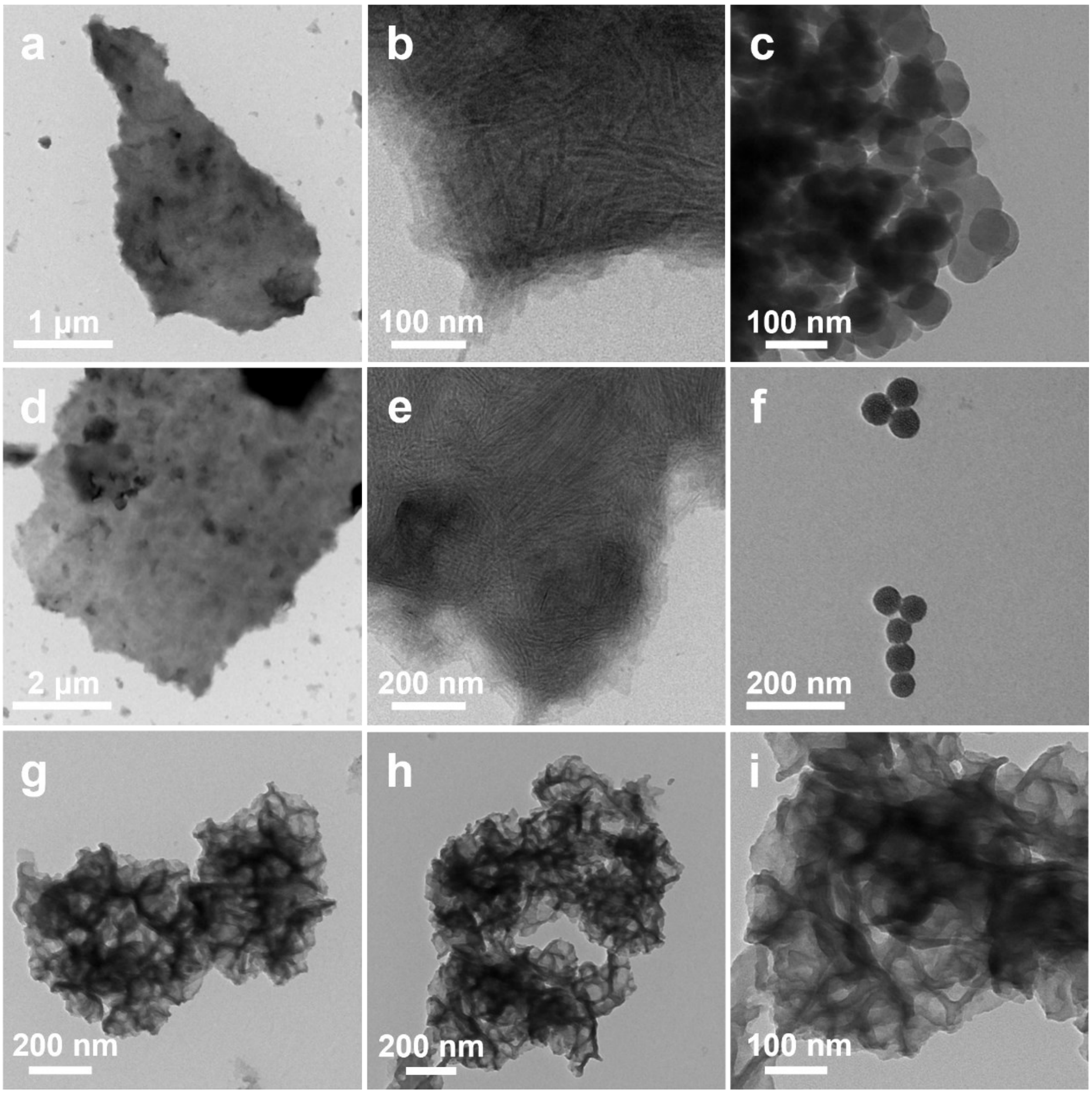

Figure S12. a–i,TEM images of the materials prepared with different volumes of NH_3 H_2O: 150 μL (a–c), 300 μL (d–f), and 600 μL (g–i).

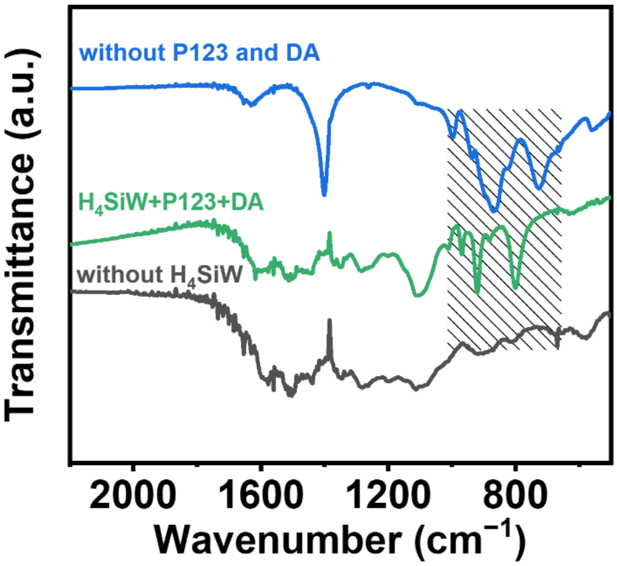

Figure S13. FT-IR spectra of the samples prepared under different conditions.

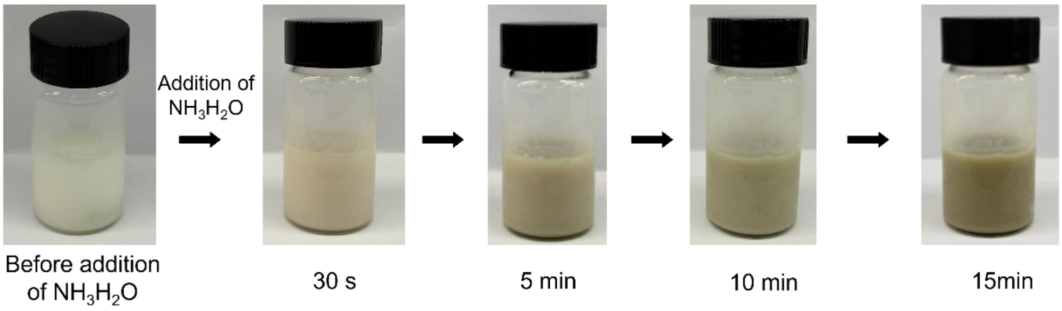

Figure S14. Photographs of the reaction mixture upon the addition of ammonium.

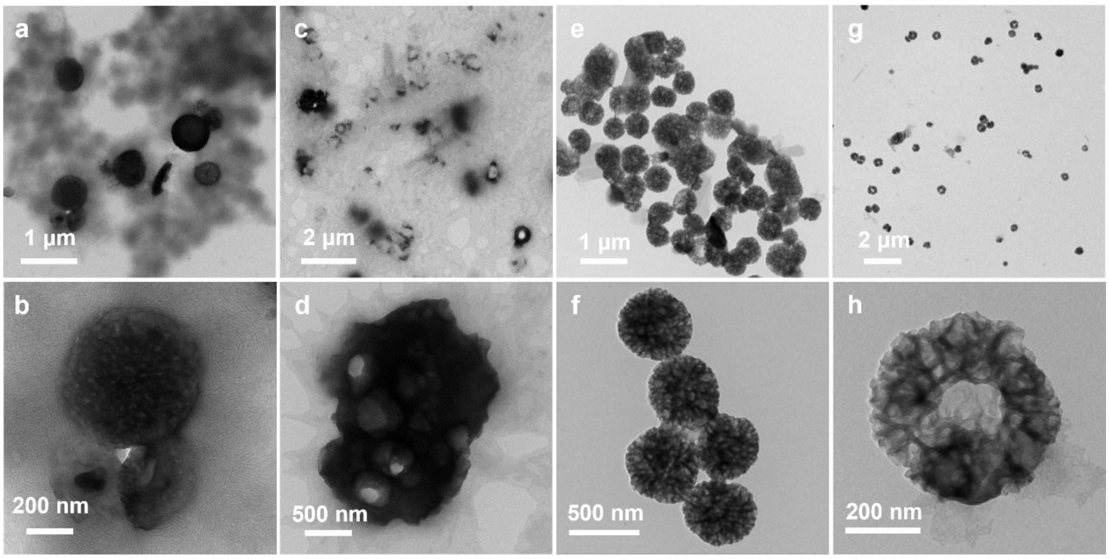

Figure S15. a–h, TEM images of HMP-0.4 prepared with different stirring rates: 0 rpm (a,b), 300 rpm (c,d), 1000 rpm (e,f), and 1500 rpm (g,h).

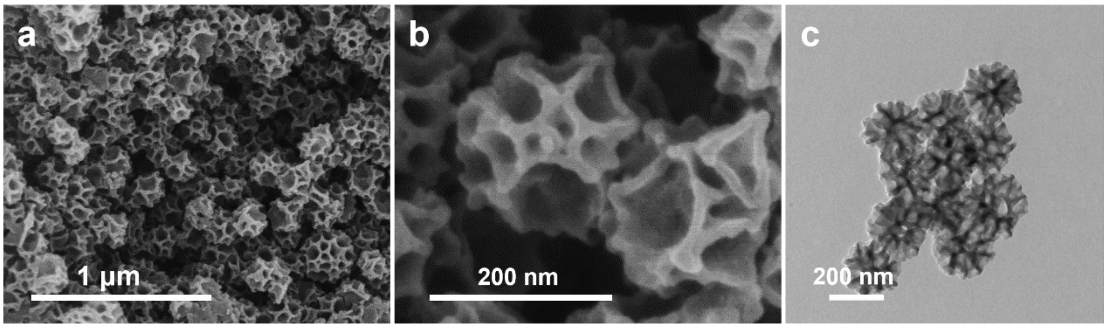

Figure S16. a–c, SEM (a,b) and TEM (c) images of MP-0.4.

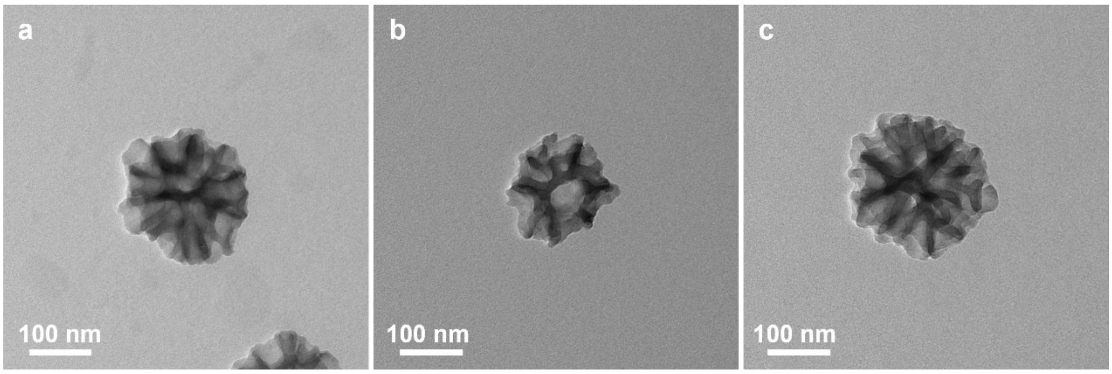

Figure S17. a–c, TEM images of the as-prepared PDA particles when replacing H_4SiW with HCl (a), HNO_3 (b), and H_2SO_4 (c).

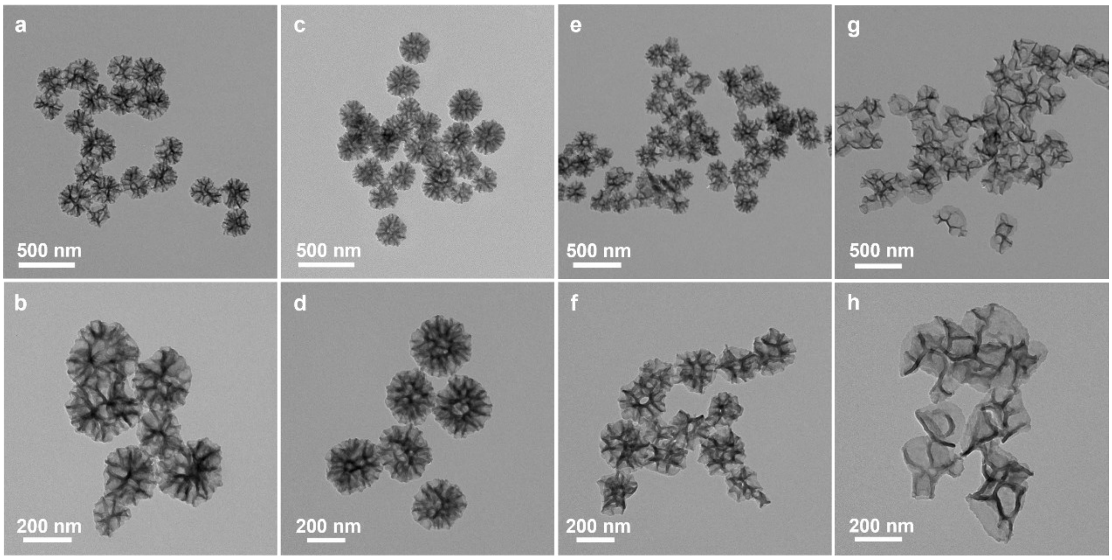

Figure S18. a–h, TEM images of the materials prepared using sulfuric acids with different pH values to replace H_4SiW: 3.0 (a,b), 1.3 (c,d), 1.0 (e,f), and 0.7 (g,h).

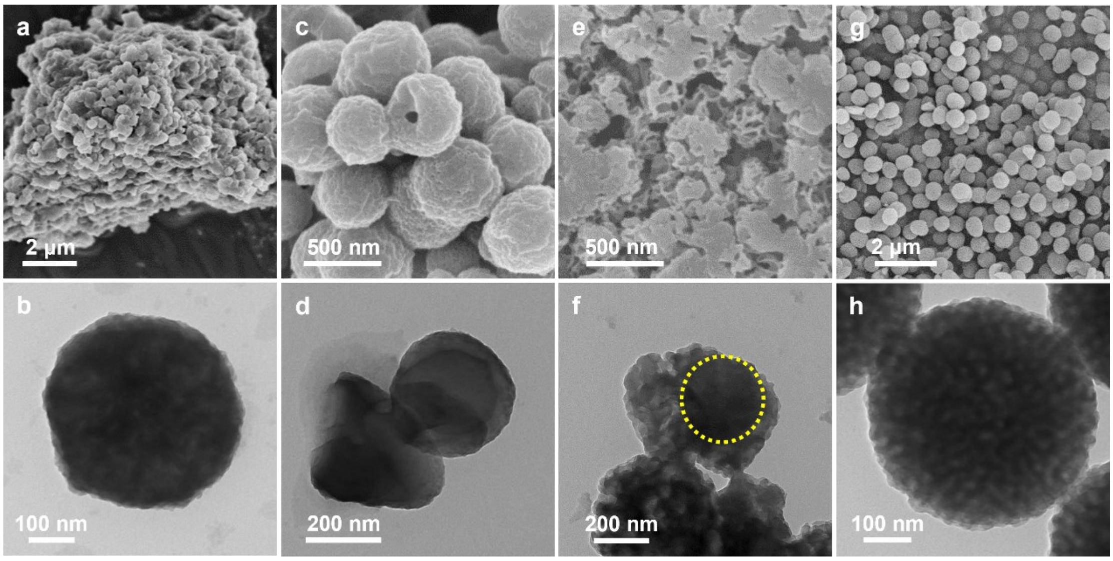

Figure S18. a–h, SEM and TEM images of the materials prepared with different volumes of TMB: 0 mL (a,b), 0.15 mL (c,d), 0.3 mL (e,f), and 0.6 mL (g,h).

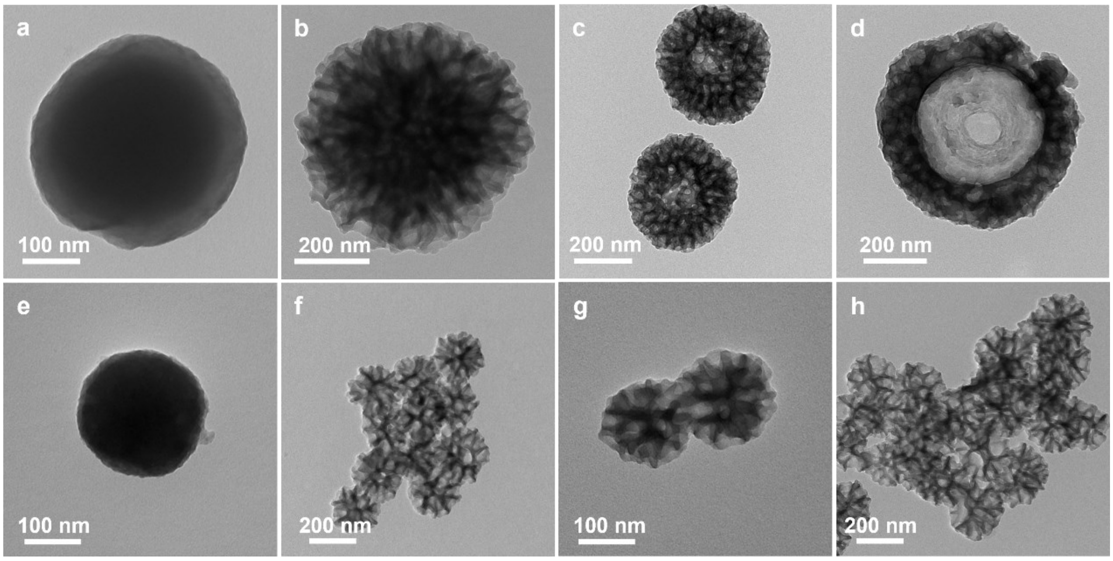

Figure S20. TEM images of (a–d) HMP-x and (e–h) MP-x obtained with different TMB volumes (x values): (a,e) 0, (b,f) 0.4, (c,g) 1.0, and (d,h) 1.5. Morphology variation is observed exclusively in HMP-x, suggesting a role of H_4SiW in morphology control.

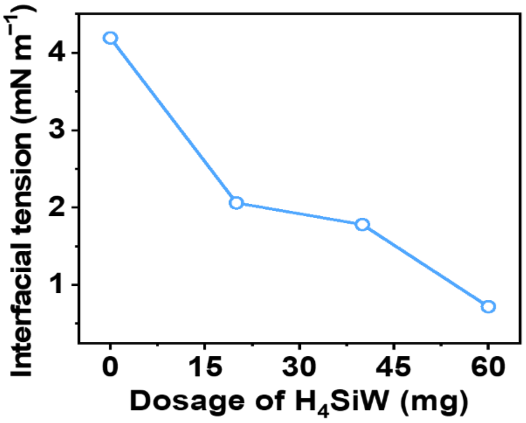

Figure S21. Interfacial tension of the emulsion system as a function of H_4SiW dosage.

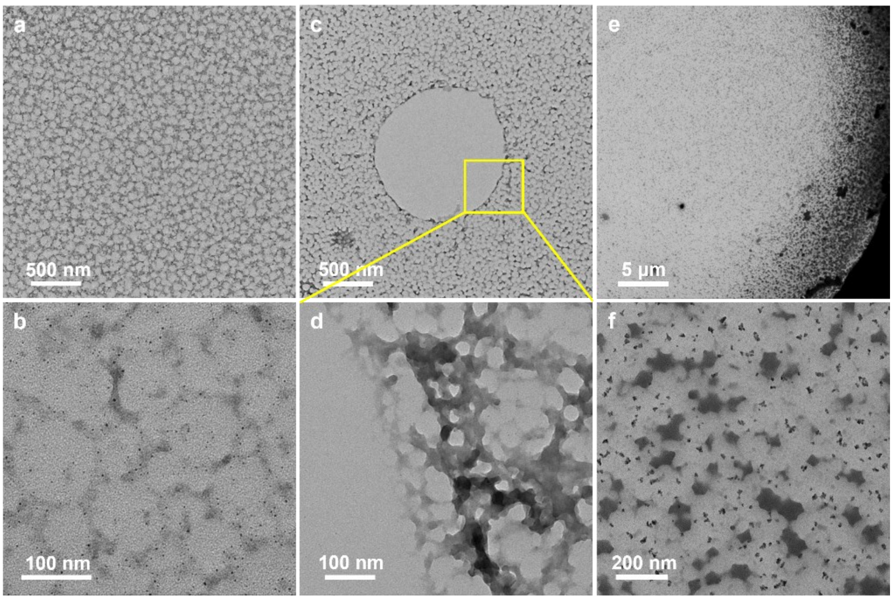

Figure S22. TEM images of the emulsion systems of MP-x before adding ammonia with x value of: 0.4 (a,b), 1.0 (c,d), and 1.5 (e,f).

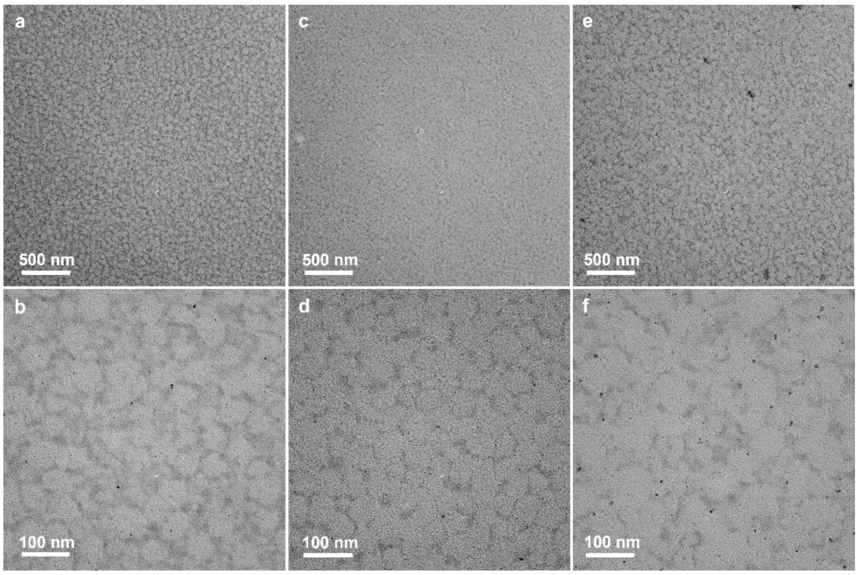

Figure S23. a–f, TEM images of the emulsion systems before adding ammonium hydroxide when replacing H_4SiW with HCl (a,b), HNO_3 (c,d), and H_2SO_4 (e,f).

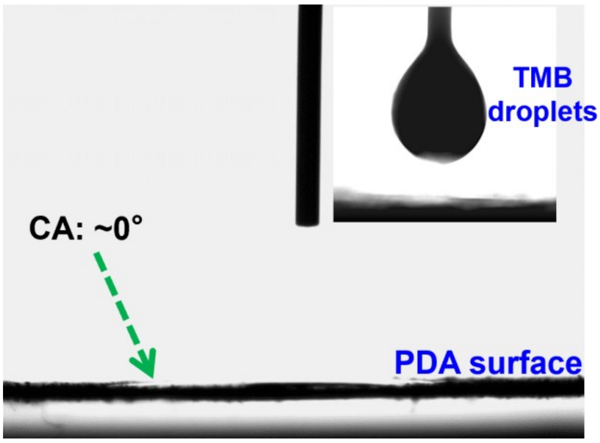

Figure S24. Photographs showing the contact angle (CA) of pure TMB on the surface of as-prepared PDA.

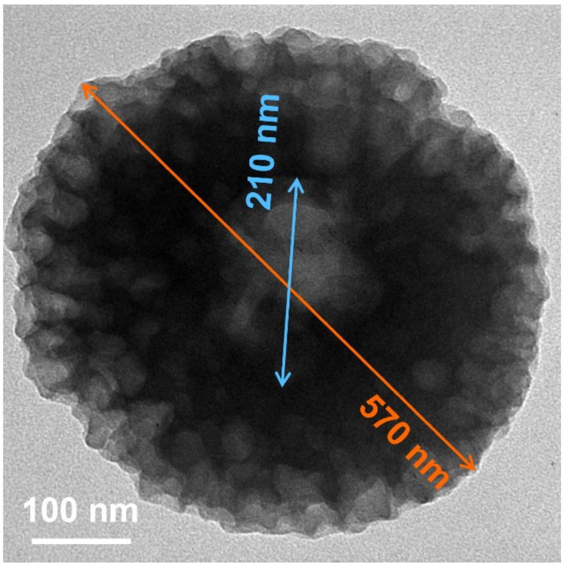

Figure S25. TEM image of a representative HMP-1.0 particle, exhibiting a cavity diameter of ~210 nm and an overall particle size of ~570 nm.

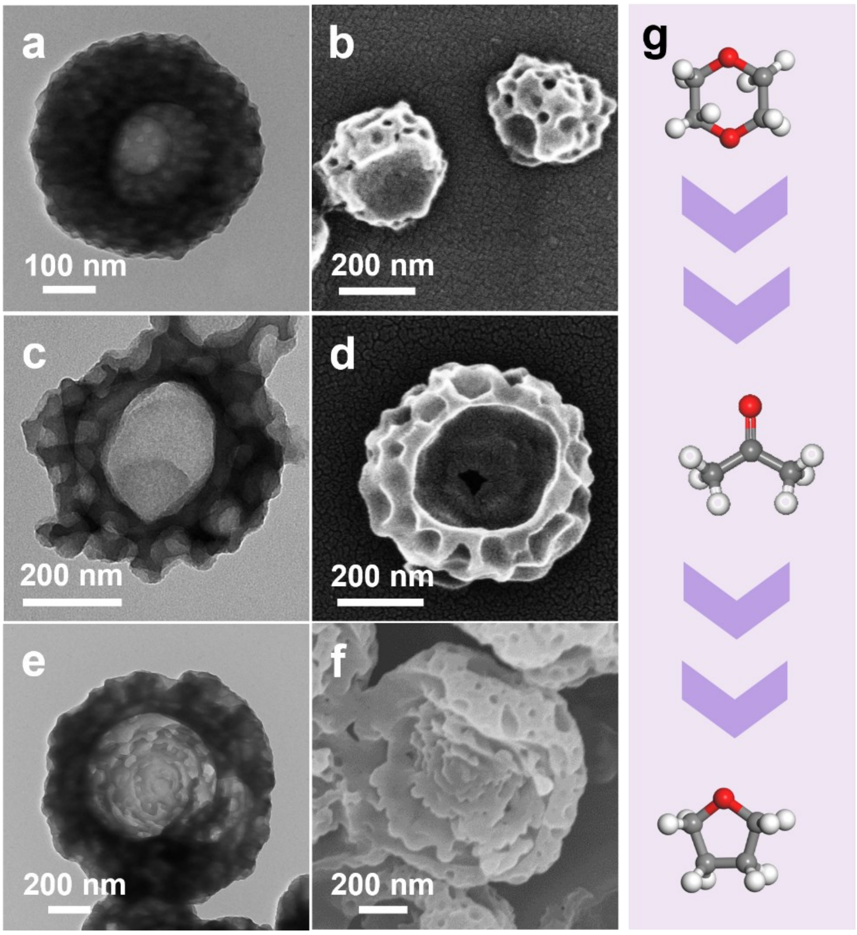

Figure S26. a–f, TEM and SEM images of the particles with different solvents to replace ethanol: (a,b) 1,4-dioxane, (c,d) acetone, (e,f) tetrahydrofuran. g, Corresponding solvent molecular structures.

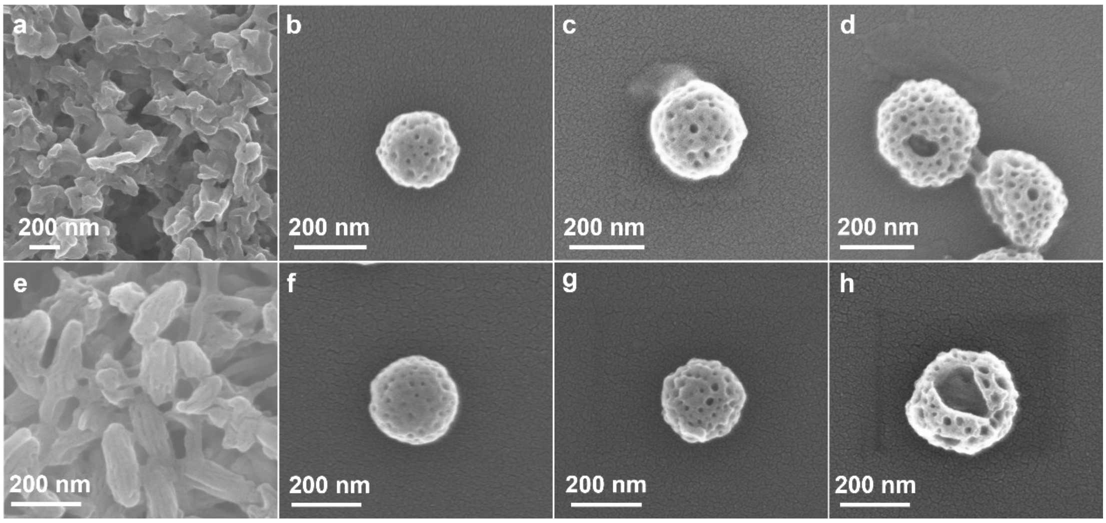

Figure S27. Samples obtained by using F127 and P123 mixtures with different mass ratios at (a) 1:0 (pure F127), (b) 1:1, (c) 2:3, and (d) 3:7. Samples obtained by using F108 and P123 mixtures with different mass ratios at (e) 1:0 (pure F108), (f) 1:1, (g) 2:3, and (h) 3:7.

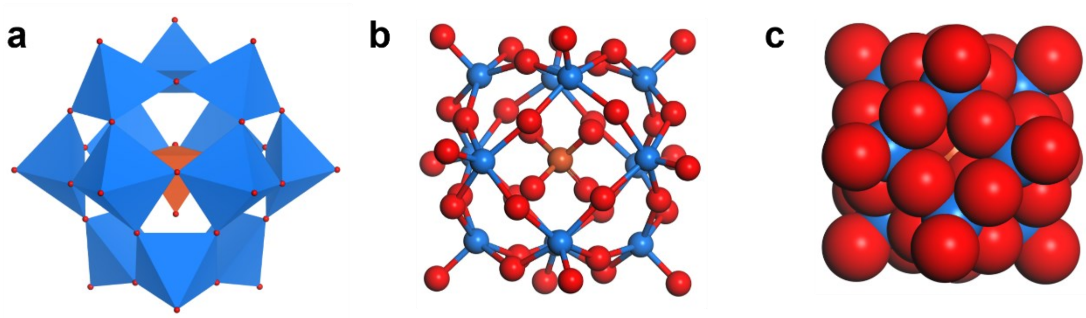

Figure S28. Several structure diagrams of a Keggin polyoxometalates anion with a general formula of $[XM_{12}O_{40}]^{n-}$, where X is the heteroatom (P^{5+}, Si^{4+}) and M is the addenda atom (Mo, W): Polyhedral model (a), Ball-and-Stick model (b), and CPK model (c).

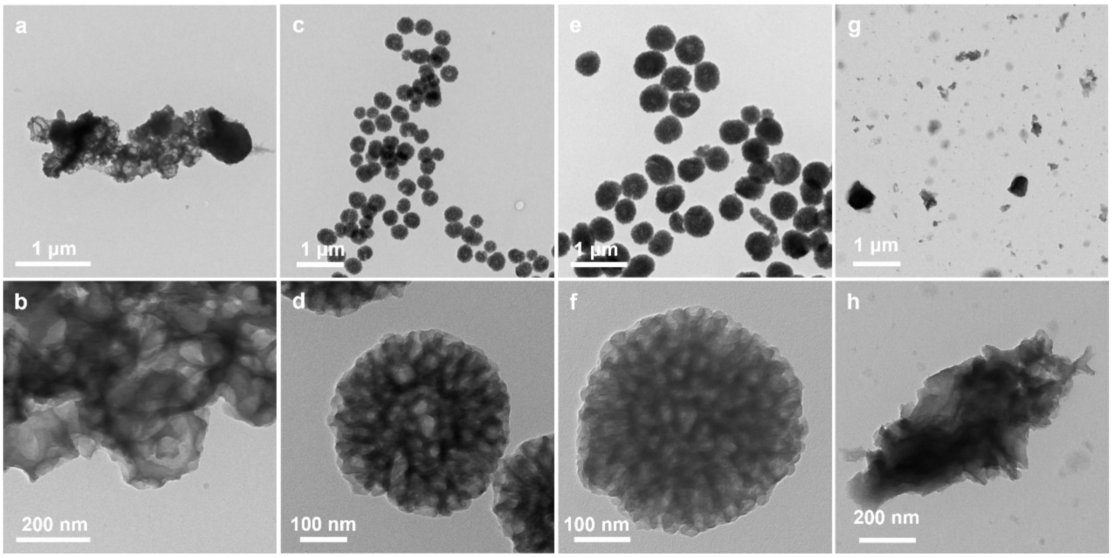

Figure S29. a–h, TEM images of the hybrid materials prepared with different amounts of H_4SiW: 20 mg (a,b), 30 mg (c,d), 40 mg (e,f), and 45 mg (g,h).

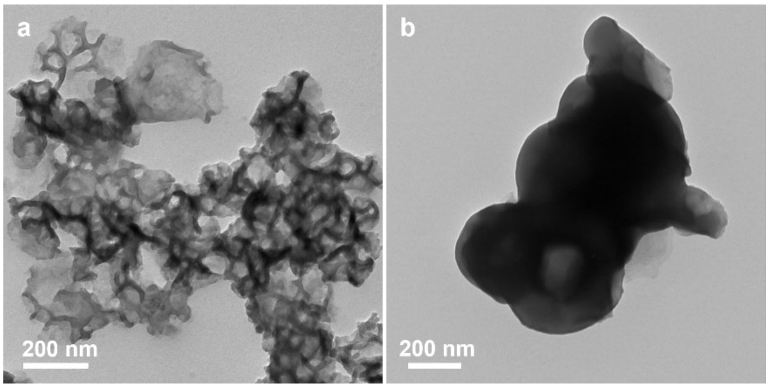

Figure S30. a,b,TEM images of the materials prepared with different amounts of P123: 0.05 g (a), 0.15 g (b).

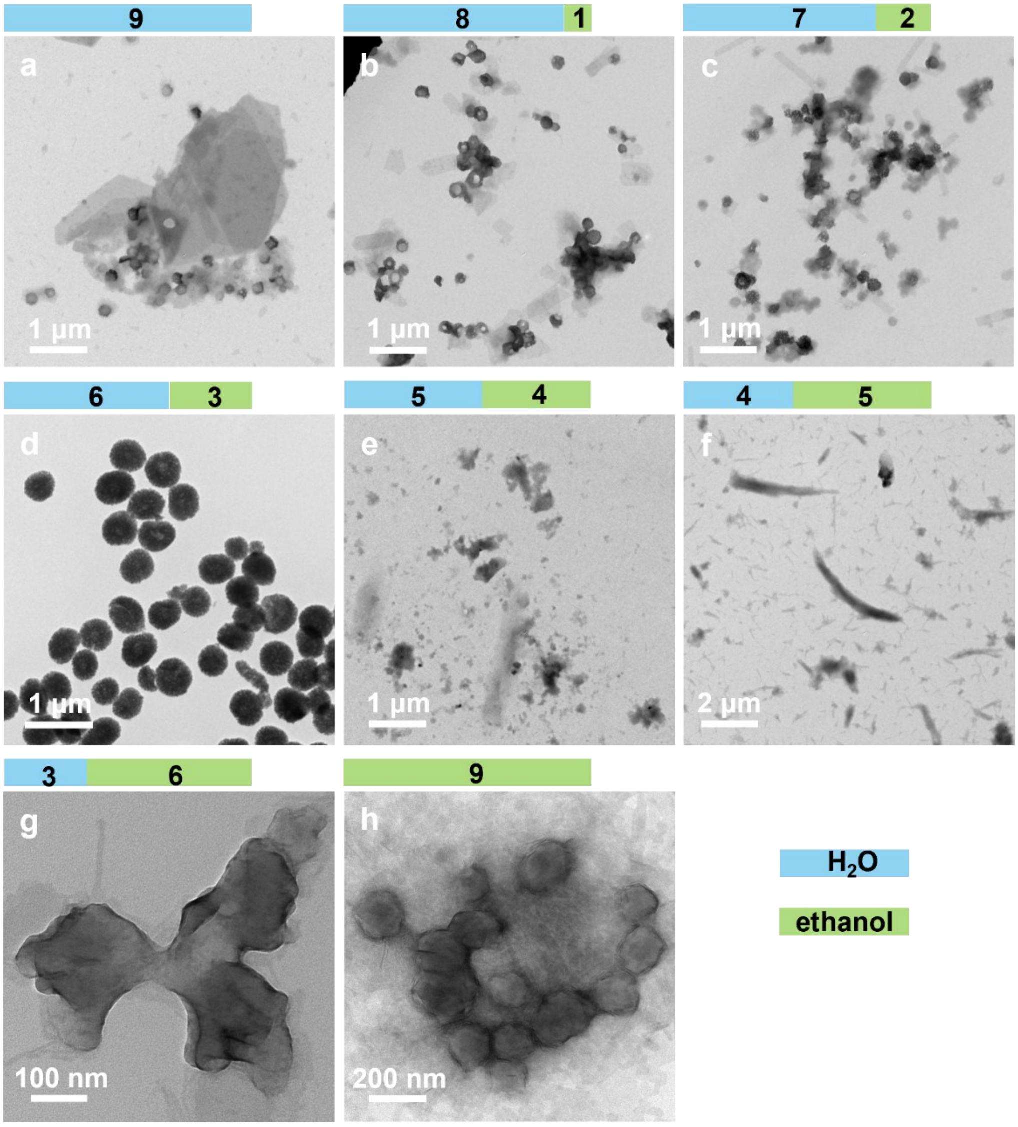

Figure S31. TEM images of the as-prepared materials with different H_2O/ethanol ratios.

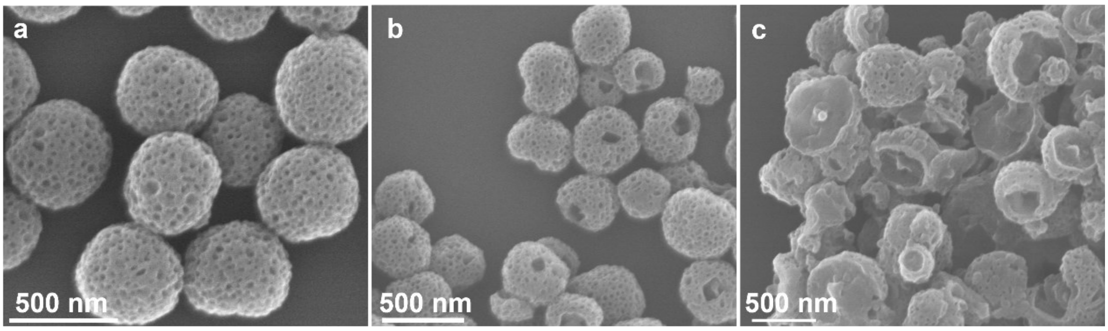

Figure S32. SEM images of (a) HMPM-0.4, (b) HMPM-1, and (d) HMPM-1.5, demonstrating tunable anisotropy.

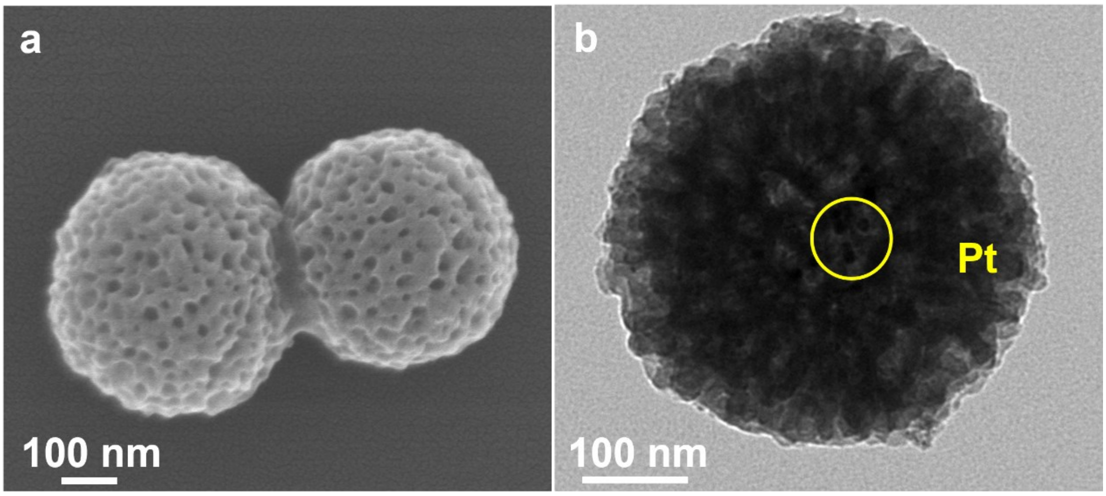

Figure S33. (a) SEM and (b) TEM images of HMPM-0.4 with Pt loading, showing that the original morphology is well preserved after Pt incorporation.

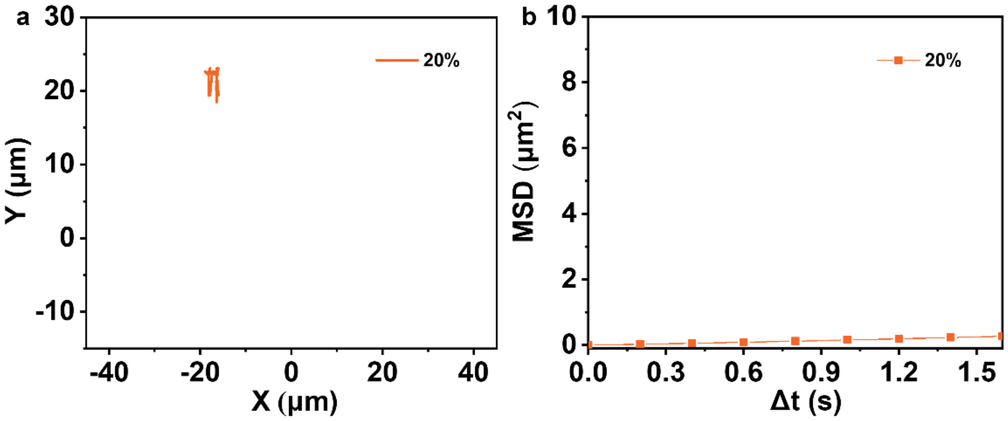

Figure S34. (a) Representative 10 s trajectory and (b) MSD curve of HMPM-0.4 in 20% H_2O_2.

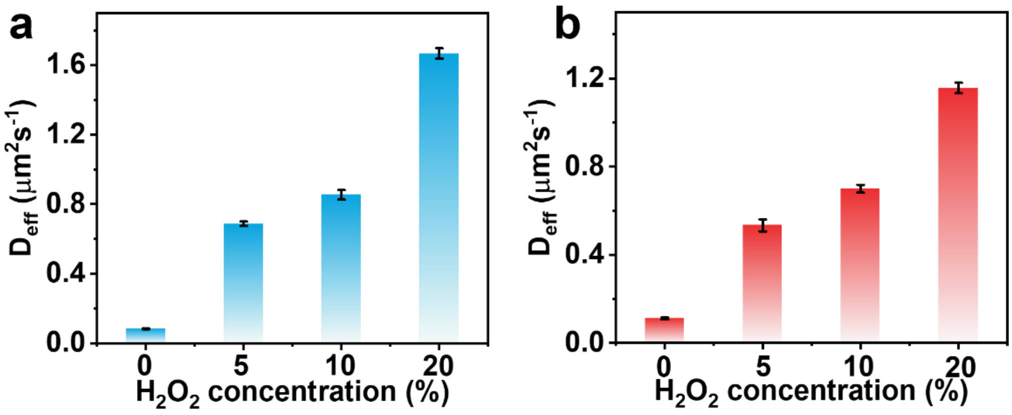

Figure S35. D_{eff} of (a) HMPM-1.0 and (b) HMPM-1.5 in hydrogen peroxide solutions with varying concentrations.

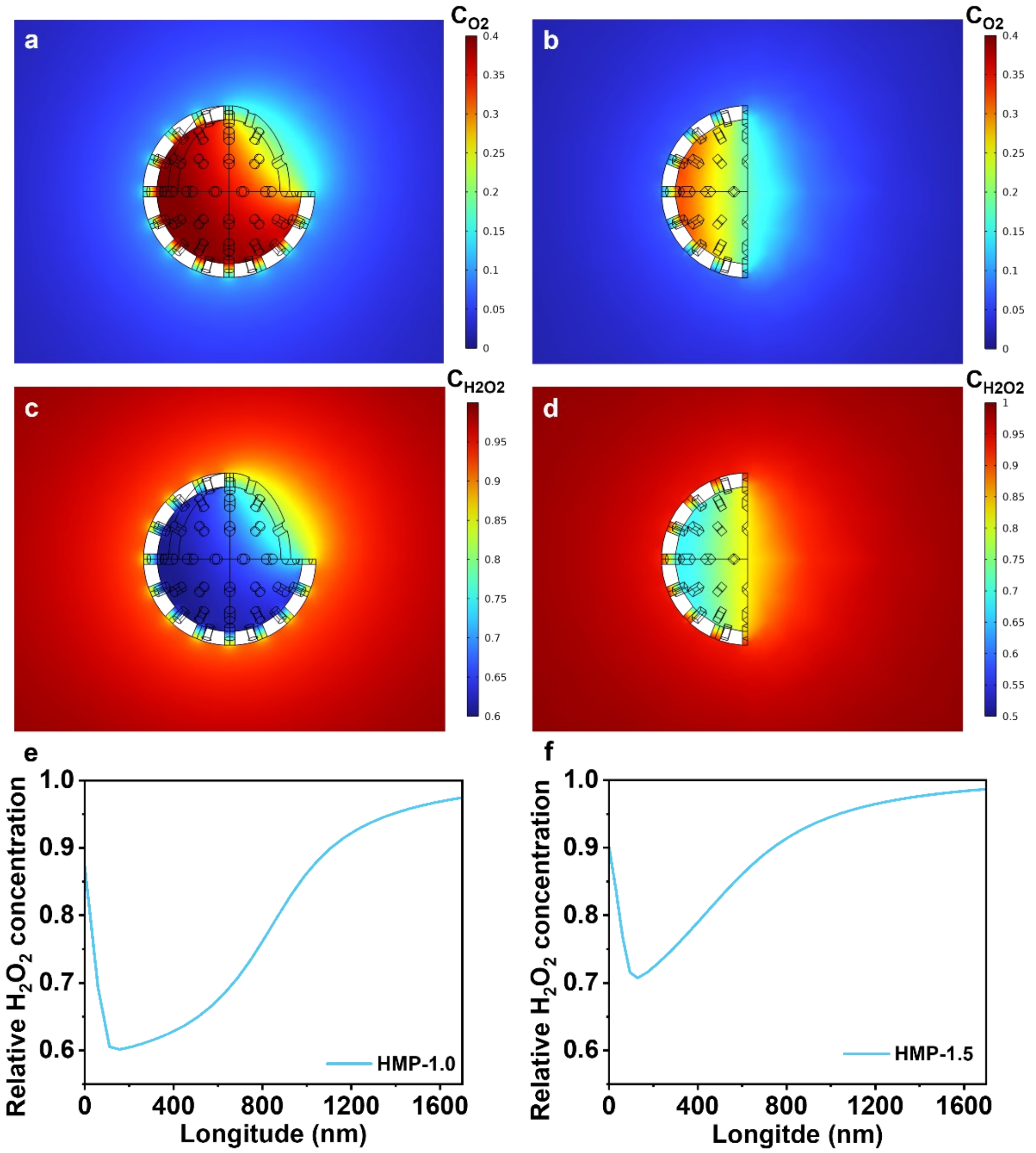

Figure S36. a,b, Simulated steady-state O_2 concentration distributions around HMPM-1.0 (a) and HMPM-1.5 (b). c,d, Simulated steady-state H_2O_2 concentration distributions around HMPM-1.0 (c) and HMPM-1.5 (d). e,f, Line profiles of the H_2O_2 concentration along the symmetry axis of HMPM-1.0 (e) and HMPM-1.5 (f).

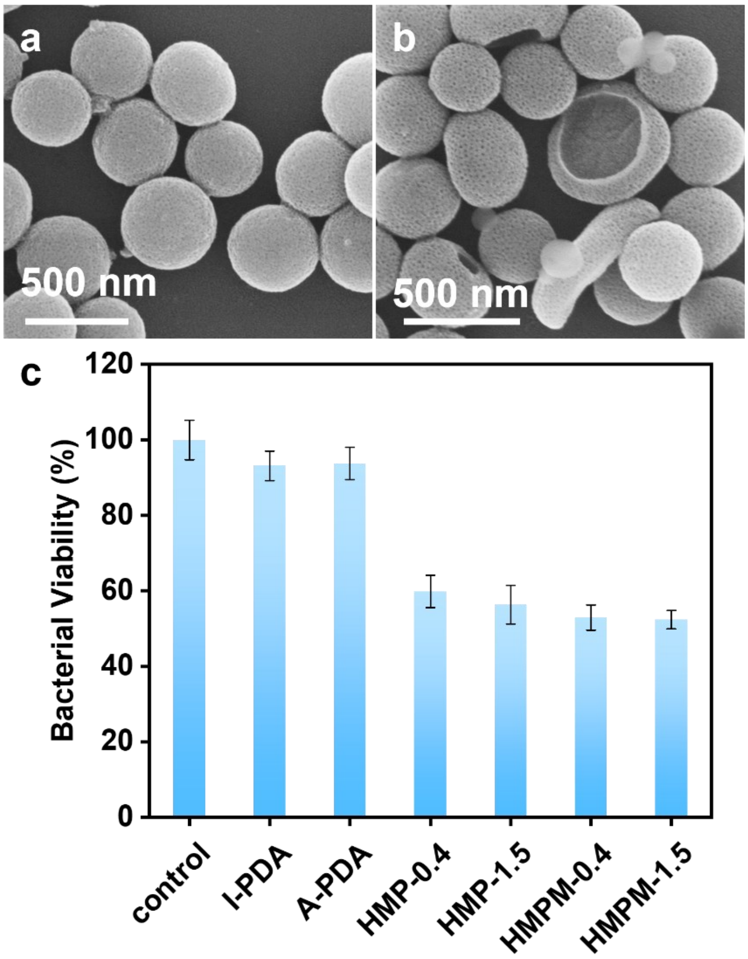

Figure S37. a,b**,** SEM images of isotropic PDA (I-PDA) and anisotropic PDA (A-PDA) nanoparticles. c, Bacterial viability of MRSA after incubation.

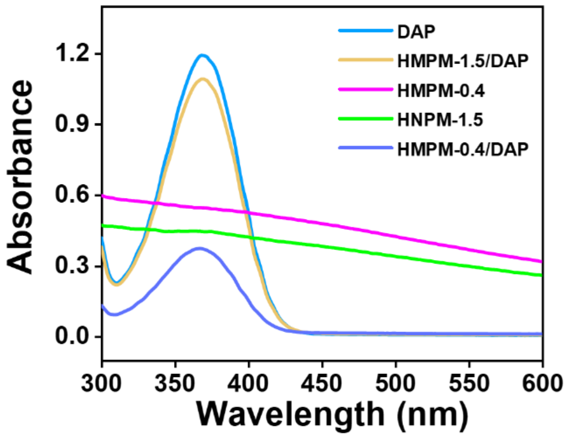

Figure S38. UV-vis absorbance spectra of different samples.

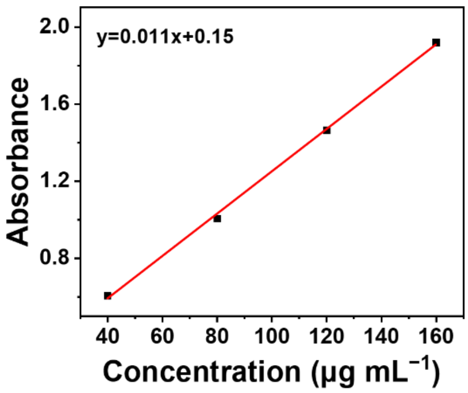

Figure S39. Standard curve of the absorbance of DAP (at 370 nm) vs concentration.

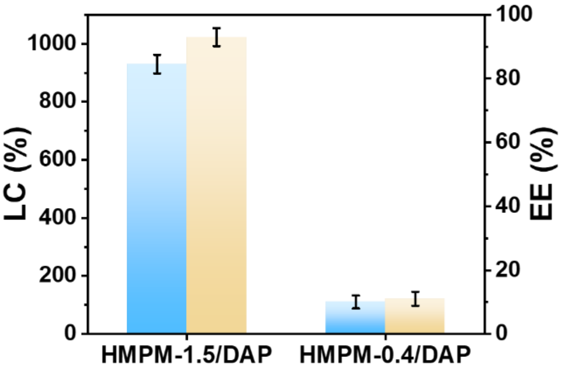

Figure S40. LC and EE of DAP on HMPM-1.5 and HMPM-0.4.

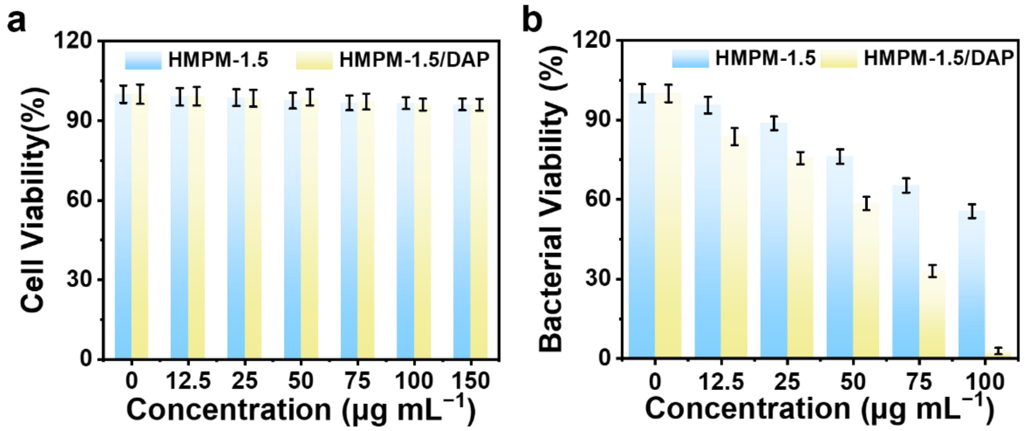

Figure S41. a, Relative cell viabilities of 3T3 cells after treatment with different concentrations (0, 12.5, 25, 50, 75, 100 and 150 μg mL^{-1}) of HMPM-1.5 and HMPM-1.5/DAP. b, Bacterial viability of MRSA after incubation with different concentrations of HMPM-1.5 and HMPM-1.5/DAP.

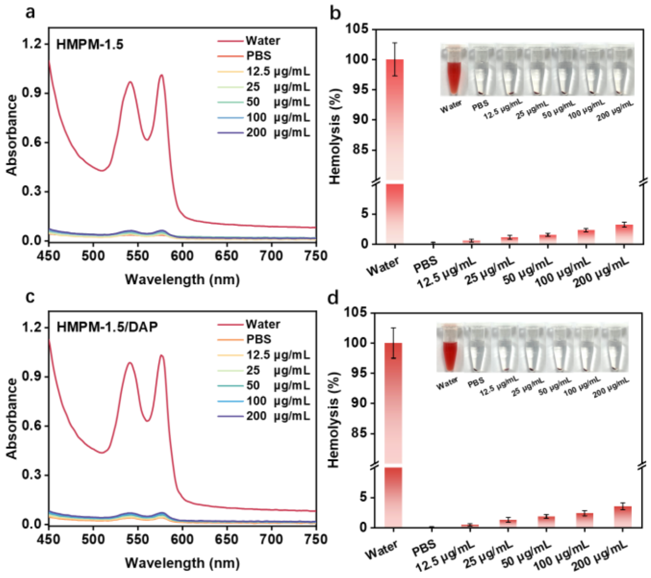

Figure S42. Hemolysis test of HMPM-1.5 (a–b) and HMPM-1.5/DAP (c–d) at various concentrations (12.5, 25, 50, 100 and 200 μg mL^{-1}).

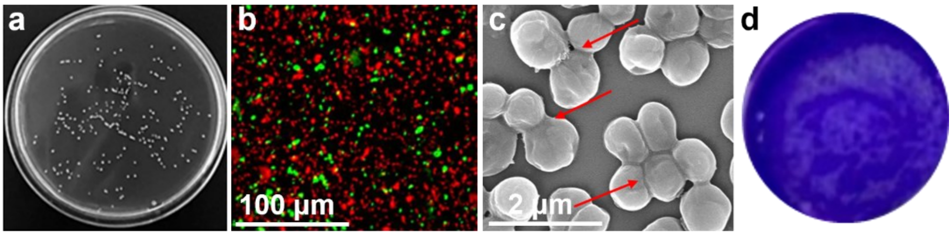

Figure S43. a–d, Photograph of colonies (a), Live/dead staining images of *MRSA* (b), SEM images of *MRSA* (c), and Crystal violet staining of *MRSA* after treatment with DAP (d).

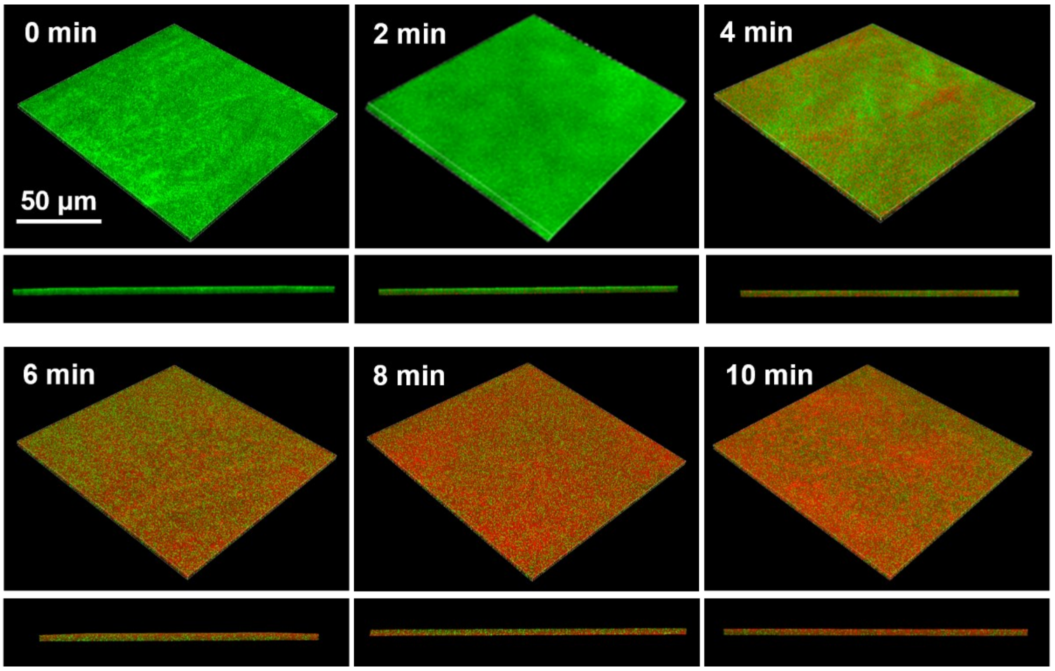

Figure S44. 3D CLSM images of biofilms treated with the HMPM-1.5 for different incubation times.

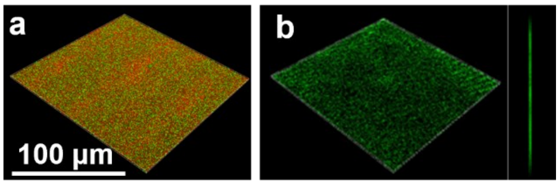

Figure S45. 3D views of SYTO 9 staining images of the biofilms after treatment with DAP

References

1. Schneider CA, Rasband WS, Eliceiri KW. NIH image to ImageJ: 25 years of image analysis. *Nat Methods* 2012; **9**: 671–5.

2. Newville M. IFEFFIT: interactive XAFS analysis and FEFF fitting. *J Synchrotron Radiat* 2001; **8**: 322–4.
